# Differential transcriptomic landscapes of multiple organs from SARS-CoV-2 early infected rhesus macaques

**DOI:** 10.1007/s13238-022-00915-5

**Published:** 2022-04-04

**Authors:** Chun-Chun Gao, Man Li, Wei Deng, Chun-Hui Ma, Yu-Sheng Chen, Yong-Qiao Sun, Tingfu Du, Qian-Lan Liu, Wen-Jie Li, Bing Zhang, Lihong Sun, Si-Meng Liu, Fengli Li, Feifei Qi, Yajin Qu, Xinyang Ge, Jiangning Liu, Peng Wang, Yamei Niu, Zhiyong Liang, Yong-Liang Zhao, Bo Huang, Xiao-Zhong Peng, Ying Yang, Chuan Qin, Wei-Min Tong, Yun-Gui Yang

**Affiliations:** 1 CAS Key Laboratory of Genomic and Precision Medicine, Collaborative Innovation Center of Genetics and Development, College of Future Technology, Beijing Institute of Genomics, Chinese Academy of Sciences, Beijing, 100101 China; 2 China National Center for Bioinformation, Beijing, 100101 China; 3 Sino-Danish College, University of Chinese Academy of Sciences, Beijing, 101408 China; 4 Department of Pathology, Beijing Ditan Hospital, Capital Medical University, Beijing, 100000 China; 5 Department of Pathology, Institute of Basic Medical Sciences, Molecular Pathology Research Center, Chinese Academy of Medical Sciences and Peking Union Medical College, Beijing, 100005 China; 6 NHC Key Laboratory of Human Disease Comparative Medicine, Beijing Key Laboratory for Animal Models of Emerging and Remerging Infectious Diseases; Institute of Laboratory Animal Science, Chinese Academy of Medical Sciences and Comparative Medicine Center, Peking Union Medical College, Beijing, 100021 China; 7 Institute of Stem Cell and Regeneration, Chinese Academy of Sciences, Beijing, 100101 China; 8 Institute of Medical Biology, Chinese Academy of Medical Sciences and Peking Union Medical College, Kunming, 650031 China; 9 Center for Experimental Animal Research, Institute of Basic Medical Sciences Chinese Academy of Medical Sciences and Peking Union Medical College, Beijing, 100005 China; 10 Department of Pathology, Peking Union Medical College Hospital, Molecular Pathology Research Center, Chinese Academy of Medical Sciences and Peking Union Medical College, Beijing, 100005 China; 11 Department of Immunology & National Key Laboratory of Medical Molecular Biology, Institute of Basic Medical Sciences, Chinese Academy of Medical Sciences and Peking Union Medical College, Beijing, 100005 China; 12 Clinical Immunology Center, Chinese Academy of Medical Sciences, Beijing, 100005 China; 13 Department of Biochemistry & Molecular Biology, Tongji Medical College, Huazhong University of Science & Technology, Wuhan, 430030 China; 14 State Key Laboratory of Medical Molecular Biology, Department of Molecular Biology and Biochemistry, Institute of Basic Medical Sciences, Medical Primate Research Center, Neuroscience Center, Chinese Academy of Medical Sciences, School of Basic Medicine Peking Union Medical College, Beijing, 100005 China

**Keywords:** SARS-CoV-2, NRP1, inflammation, central nervous system, viral encephalitis, rhesus macaque

## Abstract

SARS-CoV-2 infection causes complicated clinical manifestations with variable multi-organ injuries, however, the underlying mechanism, in particular immune responses in different organs, remains elusive. In this study, comprehensive transcriptomic alterations of 14 tissues from rhesus macaque infected with SARS-CoV-2 were analyzed. Compared to normal controls, SARS-CoV-2 infection resulted in dysregulation of genes involving diverse functions in various examined tissues/organs, with drastic transcriptomic changes in cerebral cortex and right ventricle. Intriguingly, cerebral cortex exhibited a hyperinflammatory state evidenced by significant upregulation of inflammation response-related genes. Meanwhile, expressions of coagulation, angiogenesis and fibrosis factors were also up-regulated in cerebral cortex. Based on our findings, neuropilin 1 (NRP1), a receptor of SARS-CoV-2, was significantly elevated in cerebral cortex post infection, accompanied by active immune response releasing inflammatory factors and signal transmission among tissues, which enhanced infection of the central nervous system (CNS) in a positive feedback way, leading to viral encephalitis. Overall, our study depicts a multi-tissue/organ transcriptomic landscapes of rhesus macaque with early infection of SARS-CoV-2, and provides important insights into the mechanistic basis for COVID-19-associated clinical complications.

**Supplementary Information:**

The online version contains supplementary material available at 10.1007/s13238-022-00915-5.

## Introduction

The pandemic coronavirus disease 2019 (COVID-19), caused by the severe acute respiratory syndrome coronavirus 2 (SARS-CoV-2) infection, is becoming a public health emergency and an unprecedented global threat worldwide. Together with SARS-CoV and MERS-CoV, SARS-CoV-2 belongs to the human coronavirus (CoVs) and displays a higher transmissibility than the other two CoVs. As one of the respiratory viruses, SARS-CoV-2 infection causes pneumonia and acute respiratory distress syndrome in the lungs (Zhou et al., 2020; Zhu et al., 2020). However, many infected patients had the symptoms manifested with injury in the liver (Zhang et al., 2020b), heart (Zheng et al., 2020), vascular system (Varga et al., 2020), kidney (Ronco et al., 2020), and nervous system (von Weyhern et al., 2020). The possible reason for such a multiple organ syndrome is the impeded exchange of O_2_ and CO_2_ in the virus infected lungs, leading to hypoxia and subsequent multiple organ damages. Moreover, SARS-CoV-2 could directly invade organs beyond the lungs (Bian and Team, 2020; Gupta et al., 2020). For a better clinical treatment or intervention, it is still needed to clarify whether those injuries in multiple organs are caused directly by SARS-CoV-2 infection or/and indirectly by the severe hypoxia in COVID-19 patients.

To date, a recent study based on proteomic analysis has characterized the genetic responses to SARS-CoV-2 infection in some organs of COVID-19 patient autopsies (Nie et al., 2021). However, the underlying molecular features in the infected or consequently affected organs during early stage of infection remain obscure. Here, we established the transcriptome-scale comprehensive molecular profiles of 14 tissues (cerebral cortex, brainstem, cerebellum, medulla, left ventricle, right ventricle, muscle, lung, kidney, spleen, liver, intestine, stomach and reproductive organs) collected from each of three rhesus macaques infected by SARS-CoV-2 for 7 days and one uninfected control. The dysregulated genes existed in at least one tissue of the infected rhesus macaques was counted, and a total of 18,730 genes were identified. This data resource uncovers the transcriptome-wide molecular changes in multiple organs during the early stage of SARS-CoV-2 infection, which will improve our understanding of molecular basis for the multi-organ injuries in the infected individuals.

## Results

### Wide distribution and replication of SARS-CoV-2 in organs of rhesus macaques

**Figure 1:**
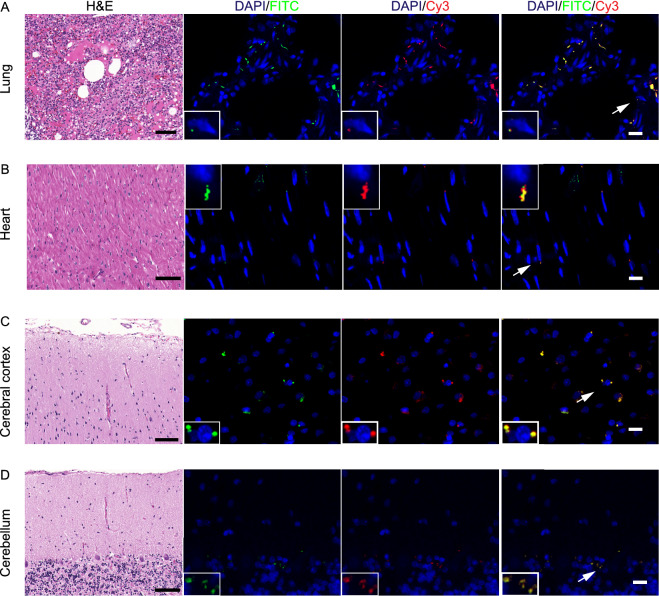
***In situ *hybridization of SARS-CoV-2 in organs of rhesus macaques**. Representative RNAscope images of SARS-CoV-2 expression (Green, FITC-labelled) and replication (Red, Cy3-labelled) in (A) lung, (B) heart, (C) cerebral cortex, (D) cerebellum from SARS-CoV-2 infected macaques of 7 dpi. Hematoxylin and Eosin (H&E) staining was used to indicate the SARS-CoV-2 *in situ* distribution and replication in each organ. Scale bars, H&E 100 μm and ISH 20 μm

SARS-CoV-2 is a positive-sense single-stranded RNA virus, and its replication is thought to occur in double-membrane vesicles, where negative-sense RNAs are produced. Based on this viral information, green and red fluorescence-labeled probes were designed to recognize sense and antisense viral RNAs, respectively. Combined with a RNAscope assay, the cellular replication of SARS-CoV-2 was well detected. Here, we used SARS-CoV-2 to infect rhesus macaque, and performed RNAscope to investigate both the *in situ* distribution and replication of SARS-CoV-2 in different tissues, including lung, heart, cerebral cortex, cerebellum, liver, kidney, microvascular vessel and testis (Figs. 1 and S1). The rhesus macaques were infected with SARS CoV-2 virus at 10^6^ TCID_50,_ and the tissues were collected at 7 days post infection (dpi) in the ABSL3 laboratory (Gao et al., 2020). Upon SARS-CoV-2 infection, a severe lung interstitial inflammation with extrudate of proteins in alveoli was evidenced in all animals examined, implying a well-established rhesus macaque model for COVID-19 (Deng et al., 2020; Yu et al., 2020b). All RNAscope analyses for expression (Green fluorescence, FITC-labelled) and replication (Red fluorescence, Cy3-labelled) of SARS-CoV-2 were performed on formalin-fixed paraffin-embedded (FFPE) sections. The fluorescence signals were acquired and analyzed using TissueGnostics. We found that in addition to the severely infected lung epithelial cells showing a focal signal for both SARS-CoV-2 expressions and replications (Fig. 1A), myocadiac cells in left and right atria and ventricles also displayed expression of SARS-CoV-2, mainly located at both side of enlarged nuclei (Fig. 1B). Likewise, abundant SARS-CoV-2 can be detected in testicular sertoli cells (Fig. S1A), probably due to their high expression of angiotensin-converting enzyme 2 (ACE2) (Tipnis et al., 2000). However, sparse signal of SARS-CoV-2 was detected in hepatocytes (Fig. S1B). In the kidney cortical regions, sparse expression and replication of SARS-CoV-2 was observed in proximal, distal convoluted tubules, and collecting tubules (Fig. S1C), as well as endothelial cells of the interstitial micro-vessels (Fig. S1D). *In suit* signal of SARS-CoV-2 was also observed in colon (Fig. S1E). No SARS-CoV-2 signal was observed in leydig and spermatocytes. Notably, we found a sparse signal of SARS-CoV-2 expression and replication in various cerebral cortex regions, including cerebral cortical neuronal cells (Fig. 1C) and cerebellar Purkinje cells (Fig. 1D). In addition, viral RNA copies were detected in most of tissues isolated from three infected macaques at 7 dpi *via* droplet digital PCR (ddPCR) (Yu et al., 2020a) (Fig. S1F; Table S1). Collectively, these data showed a wide distribution and replication of SARS-CoV-2 in multiple organs of rhesus macaques, with rich levels in the lung, heart and testis (Table S2), suggesting its higher infectivity and potential pathogenicity.

### Distinct transcriptomic features of rhesus macaque organs

**Figure. 2:**
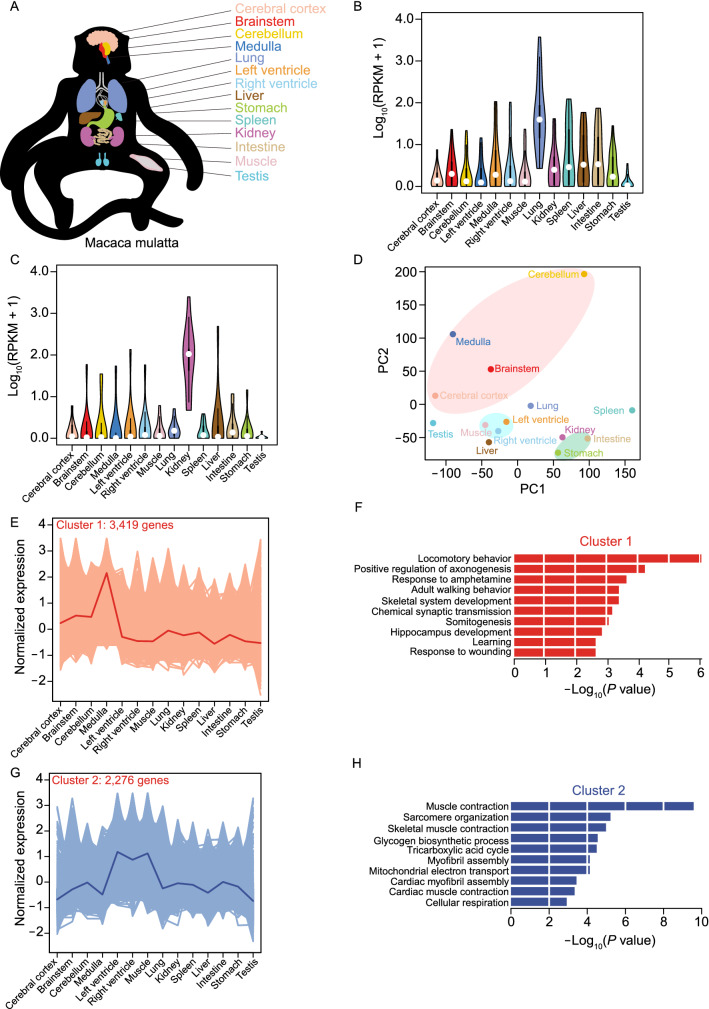
**Functional clusters of genes were identified by RNA-seq in multiple organs of rhesus macaque**. (A) Schematic diagram displaying 14 organs of rhesus macaque used in this study. (B) Violin plots showing expression profiling for specific tissue marker genes of lung in all of 14 organs. (C) Violin plots showing expression profiling for specific tissue marker genes of kidney in all of 14 organs. (D) Principal component analysis of gene expression patterns for 14 organs. (E) Line plot showing the normalized expression pattern for genes from Cluster 1, which is determined by K-means clustering analysis. The light lines represent normalized expression value for each gene from Cluster 1, while darker line represents mean of normalized expression value among all Cluster 1 genes. (F) Barplot showing the enriched GO terms for Cluster 1 genes in (E). (G) Line plot showing the normalized expression pattern for genes from Cluster 2, which is determined by K-means clustering analysis. The light lines represent normalized expression value for each gene from Cluster 2, while the darker line represents mean of normalized expression value among all Cluster 2 genes. (H) Barplot showing the enriched GO terms for Cluster 2 genes in (G)

To investigate the organ-specific transcriptomes, we obtained the transcriptomic profiles by RNA-seq in each of 14 organs from non-infected control and infected rhesus macaques (Fig. 2A; Table S3). The specific tissue markers for different organs were selected from Human Cell Landscape (Han et al., 2020b) and used to validate the data quality in the healthy individual. The markers with high expression levels in their corresponding organs were demonstrated (Figs. 2B, 2C and S2A; Table S4), justifying the integrity and specificity of our collected tissue samples. Principle components analysis (PCA) and unsupervised hierarchical clustering for different organs showed that functionally closed organs were well clustered, such as tissues of cerebral cortex, brainstem, cerebellum and medulla of the brain organ, intestine and stomach of digestive system, and left ventricle, right ventricle and muscle related cardiovascular and muscle system (Figs. 2D and S2B).

To further identify the organ-specific genes and their functions in rhesus macaque, we performed K-means clustering analysis for all tissues, and 8 clusters were categorized by their whole transcriptomic profiling (Figs. 2E, 2G and S2C–H; Table S5). The genes from different clusters displayed highly conserved enrichments in both their tissue-specific expressions and organ-related functions. For example, genes from Cluster 1 were highly expressed in the brain organ, especially medulla (Fig. 2E), and showed enrichment in nervous system related pathways, including locomotory behavior, positive regulation of axonogenesis, learning and so on (Fig. 2F), while the cardiovascular system related genes were mainly identified in Cluster 2 and shown high expression levels in heart and muscle organs (Fig. 2G and 2H). A similar enrichment mode was also observed in genes from Cluster 3 to 7, such as Cluster 3 in cerebellum with functional enrichment in synapse assembly, Cluster 4 in lung and kidney with smoothened signaling and hypoxia, Cluster 5 in liver with cholesterol metabolism, Cluster 6 in intestine with carbohydrate metabolic processing and Cluster 7 in spleen with innate immune and inflammatory responses (Fig. S2C–G and S2I–M). Moreover, genes from Cluster 8 were ubiquitously expressed in all organs (Fig. S2H) with enrichment of fundamental cellular functions, like translation and RNA processing (Fig. S2N). These results illustrated tissue/organ-specific features of whole transcriptome in gene expression and functional enrichment.

### Transcriptomic landscape of multiple tissues in SARS-CoV-2 infected rhesus macaques

To determine the SARS-CoV-2-induced transcriptional changes in 14 tissues, the tissues were collected from three rhesus macaques infected with the virus for 7 days and subjected to RNA sequencing. Based on the whole transcriptome profiles (Table S6), the infected individuals, regardless of gender, showed high transcription correlation with viral infection in each tissue (Fig. S3A). Intriguingly, cerebral cortex had a much higher difference in the transcriptomic profile than the other three brain tissues (brain stem, cerebellum and medulla). This distinct clustering result was further validated by t-distributed stochastic neighbor embedding (t-SNE) analysis (Fig. S3B), suggesting a differential response to SARS-CoV-2 infection in cerebral cortex.

**Figure 3:**
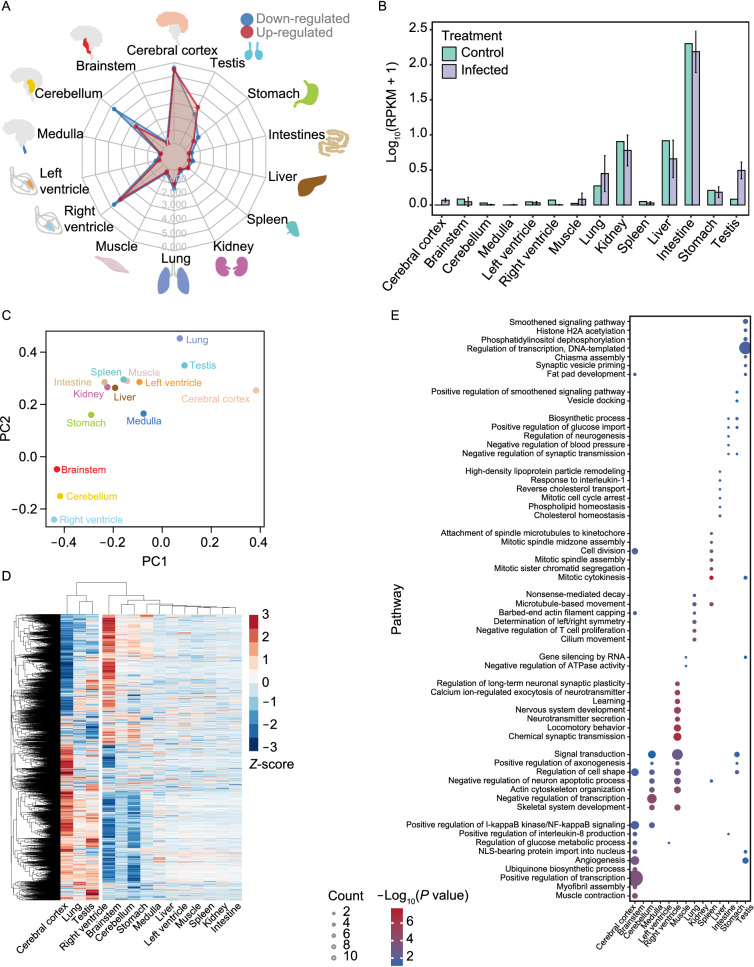
**Multi-organ transcriptomic landscape of rhesus macaques post SARS-CoV-2 infection**. (A) Radar diagram showing the numbers of dysregulated genes across multiple organs. The red points represent number of up-regulated genes in organ of SARS-CoV-2 infected rhesus macaques, while blue points stand for down-regulated genes. (B) Expression level of *ACE2* in 14 different organs for both control and infected rhesus macaques. Expression level for infected rhesus macaques is determined as mean ± standard deviation. Except testis, the standard deviations are obtained by 3 biological replicates, while testis used 2 biological replicates for only 2 infected male rhesus macaques. (C) Principal component analysis for significantly dysregulated genes, corresponding to (A), across multi-organ transcriptomes. To each organ, fold changes of gene expressions after infection are used for principal component analysis. (D) Heatmap showing the fold changes of the significantly dysregulated genes for all 14 organs. The fold change of each gene is then normalized by *Z*-score among organs. (E) Bubble chart showing the enrichment of GO terms for significantly up-regulated genes identified in each organ. Bubble size represents number of identified up-regulated genes in each term for individual organ, and *P* values from non-significance to high significance are shown as blue to red

To further evaluate the differentially expressed genes (DEGs) in multiple organs post SARS-CoV-2 infection, we compared all the infected organ samples with their corresponding controls. Since the infected rhesus macaques include 2 male and 1 female, the DEG analysis for testis was only based on 2 male infected samples. We totally identified 18,730 dysregulated genes (fold change ≥ 2 and *P* value < 0.05) among all 14 tissues and most of them were in cerebral cortex, cerebellum and right ventricle (Fig. 3A; Table S7). We further compared the expression level of *ACE2*, the first identified receptor for SARS-CoV-2 (Wrapp et al., 2020), in multi-tissues between control and infected groups (Fig. 3B). Intriguingly, among all the tissues tested, *ACE2* was highly expressed in intestine, which is consistent with previous reports (Lamers et al., 2020; Li et al., 2020), and displayed remarkably increase in lung and testis post infection, which consistent with the observations of other previous studies (Tipnis et al., 2000; Wrapp et al., 2020; Ziegler et al., 2020). Moreover, we performed both PCA and unsupervised hierarchical clustering analysis for expression changes among all dysregulated genes in 14 organs, and found that right ventricle was more closer to cerebellum and brainstem but apart from cerebral cortex (Fig. 3C and 3D), suggesting that differential signaling pathways are involved in the individual tissue/organ response to SARS-CoV-2 infection.

### Spatial response of cerebral cortex, cerebellum and right ventricle

We then performed functional enrichment analysis in Gene Ontology (GO) to determine the up- or down-regulated genes and their related functions in each organ upon SARS-CoV-2 exposure (Figs. 3E and S3C). The transcriptomic changes in each type of organ post SARS-CoV-2 infection were involved in diversely distinct functions. Interestingly, cerebellum and right ventricle shared similar transcriptomic changes in both gene dysregulation and annotated functions (Fig. 3C and 3D), such as up-regulated genes related to skeletal system development, actin cytoskeleton organization and signal transduction pathways (Fig. 3E) and down-regulated ones involving cilium assembly and telomere maintenance pathways (Fig. S3C). Beyond that, the other up-regulated genes of right ventricle were significantly annotated in chemical synaptic transmission, locomotory behavior, neurotransmitter secretion, nervous system development and learning pathways (Fig. 3E), suggesting that the homeostatic genes for nerve cells were significantly affected in right ventricle post SARS-CoV-2 infection, resulting in an enhanced secretion of neurotransmitter at early stage of viral infection. Moreover, specific transcriptomic changes in cerebral cortex post infection (Figs. 3C, 3D, S3A, and S3B) showed that the down-regulated genes were mainly related to synapse, locomotory behavior and learning terms (Fig. S3C), while up-regulated ones participated in muscle contraction, transcription, angiogenesis and nuclear factor-kappa B (NF-κB) signaling pathways (Fig. 3E). This data suggests that genes related to immune response were specifically induced in the cerebral cortex post SARS-CoV-2 infection, whereas genes functioning in the original learning and motor behavior functions were inhibited.

Relative to cerebellum and right ventricle, cerebral cortex showed an opposite tendency in dysregulated genes in terms of differential expression and functional annotation post SARS-CoV-2 infection (Figs. 3C–E and S3B–D). Moreover, the same opposite changes were also observed on those significantly dysregulated genes in cerebral cortex relative to cerebellum and right ventricle (Fig. S3E). A relatively higher proportion of intersection, especially between cerebral cortex and right ventricle, was also observed (Fig. S3F). Specifically, some metabolic process related genes were up-regulated in cerebral cortex but down-regulated in cerebellum/right ventricle (Fig. S3G), while nervous system development related genes exhibited a reverse pattern (Fig. S3H). These results suggest that brain and heart undergo drastic transcriptomic reprogramming at the early stage of SARS-CoV-2 infection.

### Elevated innate immune response of cerebral cortex

**Figure 4:**
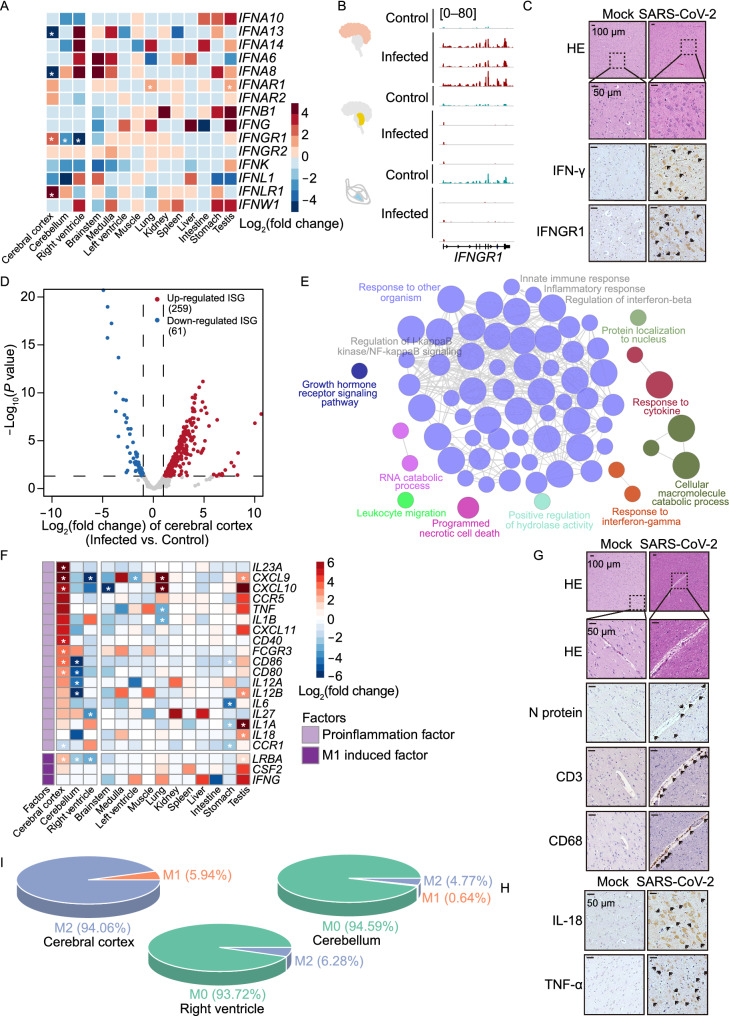
**Enhanced innate immune response in cerebral cortex after SARS-CoV-2 infection**. (A) Heatmap showing fold change of interferon genes in different organs from infected rhesus macaques compared to the control one. The white asterisk represents dysregulated genes with statistical significance (*P* value < 0.05). (B) Genome browser showing the reads abundance along *IFNGR1* in cerebral cortex, cerebellum and right ventricle from control and infected rhesus macaques. (C) HE and IHC staining showing elevated IFN-γ and its receptor IFNGR1 in cerebral cortex post SARS-CoV-2 infection. (D) Volcano plot showing the differential expression of expressed ISGs between infected and control groups in cerebral cortex. Red points: up-regulated ISGs; blue points: down-regulated ISGs. (E) Map of enriched GO functional terms for significantly up-regulated ISGs in cerebral cortex from infected rhesus macaques. Dot represents enriched GO term, while size of dot stands for its significance level (*P* value). Line means there are shared genes between two terms, while the width of lines stands for the counts of shared genes. (F) Heatmap showing the fold changes of M1 macrophage related genes after infection for each of 14 organs, individually. The fold change of each gene is then normalized by *Z*-score among organs, while asterisk represents dysregulated genes with statistical significance (*P* value < 0.05) identified in each organ. (G) Representative positive immunostaining for SARS-CoV-2 N protein, CD3^+^ T cells, and CD68^+^ macrophages. (H) Representative immuno-activities for macrophage releasing cytokines IL-18 and TNF-α in the SARS-CoV-2 infected cerebral cortex. (I) Pie chart displaying estimated proportions for different types of polarized macrophage cells. M0, original macrophage cell; M1, proinflammatory polarization macrophage; M2, anti-inflammatory polarization macrophage

Since innate immune response is the first line of defense against SARS-CoV-2 infection, which triggers the production of interferons (IFN), pro-inflammatory cytokines and chemokines (Sa Ribero et al., 2020), we further investigated the expression of interferon-related genes in each organ (Fig. 4A). Intriguingly, the dysregulated genes were enriched in cerebral cortex, cerebellum and right ventricle, and specifically, interferon gamma (IFN-γ) receptor 1 (*IFNGR1*) and interferon lambda (IFN-λ) receptor 1 (*IFNLR1*) expressions were up-regulated in the cerebral cortex (Figs. 4B and S4A), whereas *IFNGR1* showed down-regulation in the cerebellum and right ventricle (Fig. 4B). In addition, results from hematoxylin and eosin (HE) and immunohistochemical (IHC) staining assays also displayed an elevated expression of IFN-γ and its receptor IFNGR1 in the infected cerebral cortex tissue (Fig. 4C). We then focused on interferon-stimulated genes (ISGs) (Mostafavi et al., 2016) (Figs. 4D and S4B; Table S8), and observed that in cerebral cortex, a large proportion of ISGs showed elevated expression with enrichment in the pathways of response to IFN-γ and cytokine (Fig. 4E). Moreover, most of the up-regulated ISGs (112/259) in cerebral cortex showed down-regulation in both cerebellum and right ventricle (Fig. S4C) and these genes mainly enriched in type I interferon production, response to cytokine and metabolic process pathways (Fig. S4D). These findings suggest that IFN-γ related genes were specifically up-regulated in the SARS-CoV-2 infected cerebral cortex.

IFN-γ, as the first macrophage-activating factor (MAF) identified so far, facilitates immune response by promoting proinflammatory cytokine synthesis, phagocytosis and antigen-presenting capacity in activated M1 macrophages (Schreiber et al., 1985; Sica and Mantovani, 2012). To elucidate the immune response of cerebral cortex caused by IFN-γ, we evaluated the expression of inflammatory factors from polarized macrophages, and found that *IL-23*, the key factor in Th1-antigen-specific responses, as well as *CD80* and *CD86*, the co-stimulatory factors for pro-immune responses, had a significantly down-regulated in the infected cerebellum and right ventricle, but preferentially higher expression in cerebral cortex post infection (Fig. 4F). HE and IHC staining assays displayed a higher number of invaded inflammatory cells with increased infiltration of CD3^+^ T lymphocytes and CD68^+^ macrophages, and elevated inflammatory factors, IL18 and TNF-α, in infected cerebral cortex tissue (Fig. 4G and 4H). Additionally, anti-inflammation factors of M2 macrophages also showed high expression in the cortex probably for balancing the inflammatory response (Fig. S4E). To further determine the proportion of different stages of macrophages (M0: inactive macrophages, M1: pro-inflammatory macrophages, M2: anti-inflammatory macrophages), we employed CIBERSORT (Newman et al., 2015) to evaluate the amounts of immune cell types based on transcriptomic data of cerebral cortex, cerebellum and right ventricle tissues (Fig. S4F). The cerebral cortex exhibited an increased proportion of macrophages within active M1 and M2 phases upon infection, while most macrophages in cerebellum and right ventricle were in inactive M0 (Fig. 4I), suggesting that cerebral cortex harbors a specifically elevated immune response in the early stage of SARS-CoV-2 infection.

### Transcriptional regulatory network of hyperinflammation in cerebral cortex

**Figure 5:**
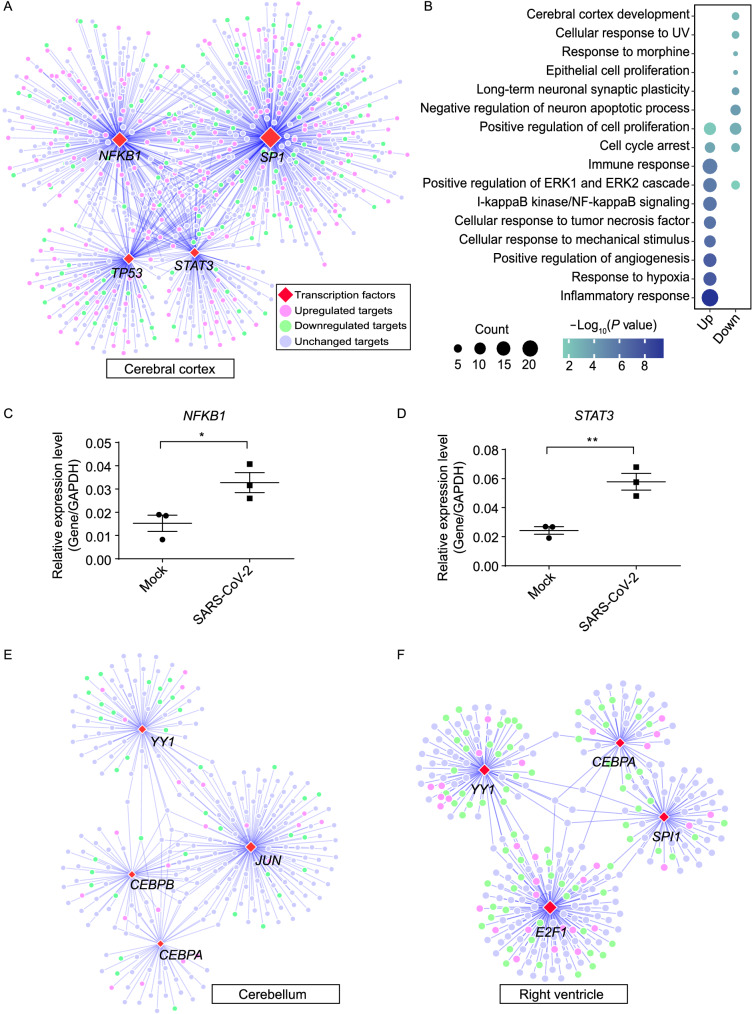
**Inflammatory transcriptional interaction network in cerebral cortex**. (A) Interaction network displaying the top four induced TFs with the most abundant targets in cerebral cortex post SARS-CoV-2 infection. Red diamonds were the induced TFs, and circular dots represented their targets. Light purple dots were up-regulated targets in cerebral cortex post infection, light green ones were down-regulated targets and light blue ones were the unchanged targets. The size of TFs represented number of their interaction pairs, and the size of targets represented their fold change post infection. (B) Bubble chart showing the enrichment of GO terms for significantly dysregulated target genes in (A). Up column represented the up-regulated targets in (A), Down column was the down-regulated targets in (A). (C and D) RT-qPCR showing higher expression of pro-inflammatory factors in cerebral cortex post SARS-CoV-2 infection. Three replicates were measured in control and SARS-CoV-2 infected samples. Asterisk represents statistical significance calculated by Student’s *t*-test, *P < 0.05 and **P < 0.01. The precise *P* values are presented in Table S14. (E) Interaction network displaying the top four induced TFs with the most abundant targets in cerebellum post SARS-CoV-2 infection. The legend was the same as (A). (F) Interaction network displaying the top four induced TFs with the most abundant targets in right ventricle post SARS-CoV-2 infection. The legend was the same as (A)

We then analyzed the signaling network regulated by transcription factors (TFs), which might associate with the susceptibility or severity of SARS-CoV-2 infection (Chen et al., 2021). Among 745 TFs identified in rhesus macaque based on transcriptional regulatory interactions of TRRUST database (Han et al., 2018), 567 showed dysregulation in the infected tissues, especially in cerebral cortex, cerebellum and right ventricle (Fig. S5A; Table S9). By matching the TF-target pairs and the dysregulated genes among these three infected tissues, we built the regulatory interaction network about the up-regulated TFs and their targets by Cytoscape (Shannon et al., 2003) (Fig. S5B–D). To further determine the infection-elicited TF network, four up-regulated TFs with their most interacting targets were retrieved to build a relatively simple regulatory interaction network for cerebral cortex (Fig. 5A), cerebellum (Fig. 5E) and right ventricle (Fig. 5F). The up-regulated targets in cerebral cortex post infection were significantly annotated in response pathways to inflammation and hypoxia (Fig. 5B), and the elevated proinflammatory factors were also observed in the infected cerebral cortex tissues by quantitative reverse-transcription PCR (RT-qPCR) (Fig. 5C and 5D). These findings suggest that SARS-CoV-2 might hijack the host transcriptional regulatory mechanism to induce hyper-inflammatory state *via* TFs in cerebral cortex, but not in cerebellum and right ventricle.

### Hypercytokinemia, thrombosis, angiogenesis and fibrotic factors elevated in cerebral cortex

The hallmark of COVID-19 pathogenesis is the cytokine storm with higher levels of proinflammatory cytokines, such as interleukin-1β (IL-1β), interleukin-2 (IL-2), interleukin-6 (IL-6), tumor necrosis factor (TNF-α), granulocyte-macrophage colony-stimulating factor (GM-CSF), chemokines (C-C-motif chemokine ligand (CCL)-2, CCL-3 and CCL-5), also interleukin-2 (IL-2), interleukin-7 (IL-7) and, interleukin-10 (IL-10) (Ruan et al., 2020). The severe cases with hyperinflammatory syndrome can include coagulation impairment (Han et al., 2020a), abnormal angiogenesis (Ackermann et al., 2020), cystic fibrosis (Fainardi et al., 2020) and even death (Ruan et al., 2020; Wang et al., 2020). We then examined the expression of those factors using the transcriptomic data from various tissues of rhesus macaques, and observed their dysregulation in the early stage of infection, especially in cerebral cortex, cerebellum and right ventricle (Fig. S6A; Table S10).

**Figure 6:**
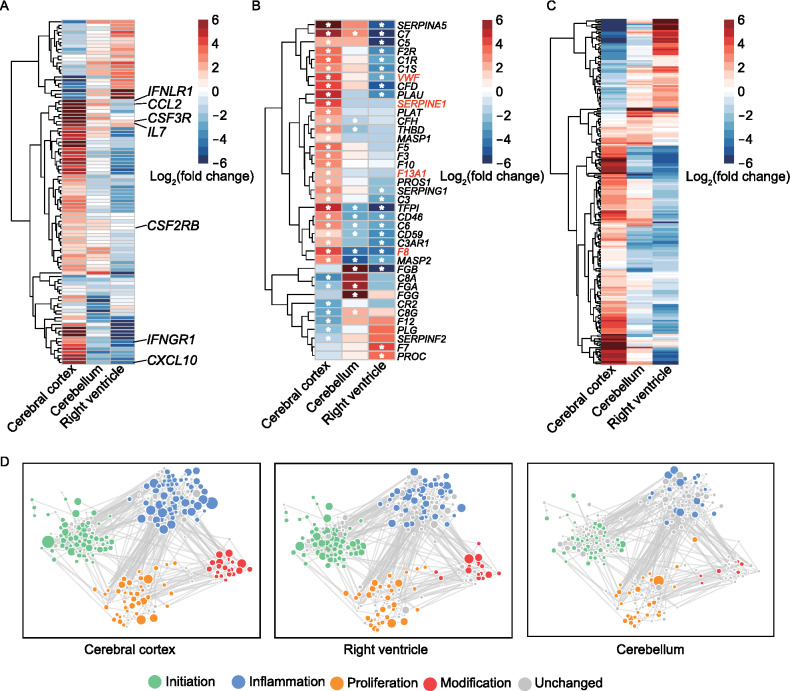
**Factors of cytokine, coagulation, angiogenesis and fibrosis significantly induced in cerebral cortex post infection**. (A) Heatmap showing fold change of dysregulated cytokines among cerebral cortex, cerebellum and right ventricle organs from infected rhesus macaques compared to the control one. (B) Heatmap showing fold change of dysregulated coagulation factors among cerebral cortex, cerebellum and right ventricle organs from infected rhesus macaques compared to the control one. The white asterisk represents dysregulated genes with statistical significance (*P* value < 0.05). (C) Heatmap showing fold change of dysregulated angiogenesis factors among cerebral cortex, cerebellum and right ventricle organs from infected rhesus macaques compared to the control one. (D) Interaction network showing the stage-specific dysregulated fibrotic genes in cerebral cortex (left), right ventricle (middle) and cerebellum (right) post SARS-CoV-2 infection over four fibrosis stages, including initiation (green), inflammation (light blue), proliferation (orange) and modification (red). The interactions were built based on the STRING database using Cytoscape. The size of bubbles represented the value of fold change of dysregulated genes from infected rhesus macaques compared to the control one

The cytokines retrieved from M9809 gene set of Molecular Signatures Database in Gene Set Enrichment Analysis (GSEA) (Subramanian et al., 2005), such as *IL-7*, *CCL2*, *CXCL10* and *IFNs*, were significantly up-regulated in cerebral cortex, but markedly down-regulated in cerebellum and right ventricle post infection (Fig. 6A). Moreover, these cytokines were enriched in the pathways of cytokine-mediated signaling pathway and acute inflammatory response (Fig. S6B), suggesting a state of hypercytokinemia or cytokine storm in the cerebral cortex. In addition, among the coagulation factors retrieved from gene set of GSEA, over half of them were dysregulated, and showed the most significant changes in three aforementioned tissues. Specifically, the coagulation factors with significant upregulation in cerebral cortex include coagulation factors prothrombin XIIIa (F13A1) (Muszbek et al., 2011; Peyvandi et al., 2011), glycoprotein VWF binding to factor VIII (F8) (Muszbek et al., 2011; Peyvandi et al., 2011) and plasminogen activator inhibitor 1 (SERPINE1) (Chapin and Hajjar, 2015) (Fig. 6B). These up-regulated cytokines were annotated in blood coagulation pathway (Fig. S6C), indicating an impaired coagulation in cerebral cortex. Similarly, most angiogenesis factors, annotated in nCounter PanCancer Progression Panel (NanoString Technologies), were aberrantly regulated with significant changes in cerebral cortex relative to cerebellum and right ventricle (Fig. 6C), and enriched in angiogenesis and some signaling pathways (Fig. S6D). suggesting an abnormal angiogenetic state in the virus infected cerebral cortex.

Fibrosis is usually divided into four stages: initiation is triggered by a cascade of immune response and stress, inflammation with activated inflammatory signaling pathways, proliferation with differentiation and proliferation of fibroblasts, and modification with restructured extracellular matrix (ECM) composed of immune cells and fibroblasts. We have quantified the expression of 771 fibrosis-related genes from mCounter Fibrosis Panel (NanoString Technologies), and found that 567 genes were dysregulated in at least one tissue post viral infection. We built an interaction network from String database (Szklarczyk et al., 2019) using these altered genes by Cytoscape (Shannon et al., 2003) (Figs. 6D and S6E). Dysregulated fibrosis markers were dominant in cerebral cortex, cerebellum and right ventricle, while inflammatory fibrosis markers were significantly dysregulated in cerebral cortex (Fig. 6D), revealing an abnormal fibrotic response in cerebral cortex at the early stage of SARS-CoV-2 infection.

Thus, SARS-CoV-2 infection rapidly induces the abnormal state of hypercytokinemia, thrombosis, angiogenesis and fibrosis, in particular the cerebral cortex. As these imbalanced activities could facilitate the formation of microthrombi (Palta et al., 2014), SARS-CoV-2 patients have higher risk to develop microthrombi in brain (Wichmann, 2020; Wichmann et al., 2020) and other systems as well (Zheng et al., 2020).

### NRP1 and signaling transmission may enhance the CNS infection

**Figure 7:**
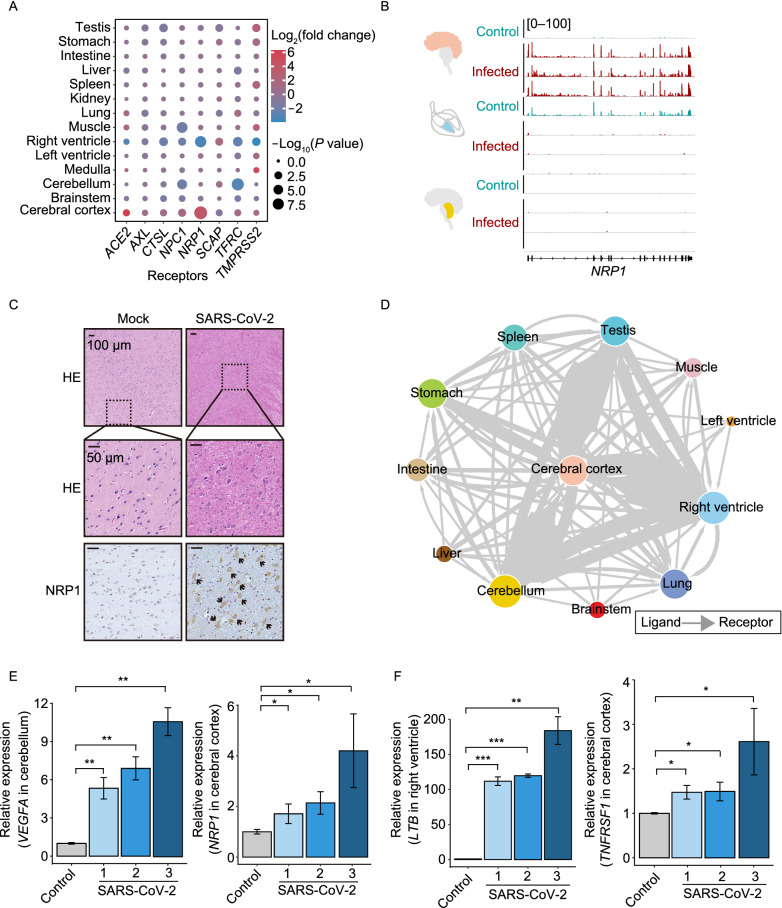
**Neuronal receptor and signaling transmission pathways enhance the cerebral cortex infection**. (A) Bubble chart showing the fold change of the identified receptors for SARS-CoV-2 infection from infected rhesus macaques compared to the control one. (B) Genome browser showing the reads abundance along *NRP1* in cerebral cortex, right ventricle and cerebellum from control and infected rhesus macaques. (C) HE and IHC staining showing induced NRP1 in neurons of cerebral cortex post SARS-CoV-2 infection. (D) Interaction network of multiple organs displaying the tissues communication based on the ligand-receptor pairs. The size of nodes represented the number of significantly induced ligands or receptors post SARS-CoV-2 infection in each organ, the direction of arrows was the signaling transduction from ligand to receptor between two organs, and the width of arrows were the number of significantly induced ligand-receptor pairs post infection between two organs. (E and F) RT-qPCR analysis showing that *VEGFA* (E, left) and *LTB* (F, left) as ligands were significantly up-regulated in cerebellum and right ventricle of SARS-CoV-2 infected macaques, respectively. Their paired receptors *NRP1* (E, right) and *TNFRSF1* (F, right) also displayed significantly induced expression in cerebral cortex after infection. Three replicates were measured in each tissue sample. Asterisk represents statistical significance calculated by Student’s *t*-test, *P < 0.05, **P < 0.01 and ***P < 0.001. The precise *P* values are presented in Table S14

We next examined the expression of reported receptors (Cantuti-Castelvetri et al., 2020; Daly et al., 2020; Tang et al., 2020; Ziegler et al., 2020; Vial et al., 2021; Wang et al., 2021b) for SARS-CoV-2 in multi-tissues post infection and observed that Neuropilin-1 (*NRP1*) was significantly up-regulated in cerebral cortex (Fig. 7A and 7B). Moreover, HE and IHC staining assays also showed elevated NRP1 in the infected cerebral cortex tissues (Fig. 7C). It has been reported that NRP1 expression is higher in nasal epithelium rendering susceptible to SARS-CoV-2 infection (Cantuti-Castelvetri et al., 2020; Daly et al., 2020). It is likely that SARS-CoV-2 may enter the cerebral tissues by crossing the blood-brain barrier *via* an upper nasal transcribrial route (Dey et al., 2021). It is worth noting that *NRP1* had been reported to be ubiquitously expressed in the brain regions, especially in hippocampal formation (Davies et al., 2020) and showed an elevated expression in the infected olfactory epithelial cells isolated from human COVID-19 autopsies (Cantuti-Castelvetri et al., 2020). Our findings, in corroboration with previous report (Gudowska-Sawczuk and Mroczko, 2021), suggest that NRP1 may be up-regulated in naïve immune response, and serves as a receptor to enhance the entry of SARS-CoV-2 into the central nervous system (CNS).

To elucidate the potential interactions among organs post infection, we used the ligand-receptor pairs from CellTalkDB (Shao et al., 2020) to examine their transcriptomic changes in the infected organs, and observed a close communication between cerebral cortex and right ventricle/cerebellum (Fig. 7D; Table S11) with higher expression of ligands in cerebellum and right ventricle and its corresponding receptors in cerebral cortex (Figs. 7E, 7F and S7A). Those genes were mainly involved in the regulation of cell migration and axon guidance pathways (Fig. S7B), which is consistent with the findings of significantly up-regulated genes in these two organs (Fig. 3E). Recent studies have shown that vascular leakage and compromised blood-brain barrier are linked with neuroinflammation of COVID-19 patients (Schwabenland et al., 2021). Collectively, these results suggest another plausible mechanism through which the signaling molecules secreted by right ventricle and cerebellum could transmit to cerebral cortex through the blood circulation system to target the receptors and enhance the responses to SARS-CoV-2 infection (Fig. S7C).

## Discussion

In this study, we established a transcriptomic atlas of 14 tissues collected from rhesus macaque infected with SARS-CoV-2 at 7 dpi. Through analyzing and comparing the dysregulated genes among various tissues, we observed the following typical features: (1) widespread and replication of SARS-CoV-2 in multiple tissues; (2) tissue/organ-specific responses and functional differences after virus infection; (3) innate immune response is enhanced in the cerebral cortex with elevated levels of cytokines, coagulation, angiogenesis and fibrotic factors post SARS-CoV-2 infection; (4) hyperinflammatory state in the cerebral cortex; (5) NRP1 may enhance the entry of SARS-CoV-2 into the central nervous system (CNS); (6) cytokines secreted by both macrophages and activated T cells contribute to the inflammatory encephalopathy caused by SARS-CoV-2 infection. Our study provides the transcriptome profiling of various tissues from the living primate host post SARS-CoV-2 infection, and illustrates that wide-spread transcriptomic changes involving diverse functions exist in multi-organs, and an elevated immune response in cerebral cortex was the prominent feature at the early stage of SARS-CoV-2 infection.

Similar to the epidemic of SARS, COVID-19 patients often present with respiratory-like illnesses with highly chances to progress to severe pneumonia, suggesting that the lung is the primary tropism of SARS-CoV-2 (Peiris et al., 2003), even though accumulative studies reported that COVID-19 patients also suffer from injuries of liver, heart, kidney, and so on (Kudose et al., 2020; Tian et al., 2020; Wichmann et al., 2020; Yang et al., 2020). However, systemic study about the effect of SARS-CoV-2 on multi-organs, especially nerve system and cardiovascular system in living host, is still lacking till recently. The tissue tropism of SARS-CoV-2 depends on susceptibility and permissiveness in a specific host cell. ACE2, as the primary cellular receptor for SARS-CoV-2, has been reported to be highly expressed in various organ types, including intestine and lung (Lamers et al., 2020; Li et al., 2020; Wrapp et al., 2020; Ziegler et al., 2020), but extremely low expression in the brain tissues (Kim et al., 2014; Giordo et al., 2021). Consistently, we also observed an enhanced *ACE2* expression in lung after SARS-CoV-2 infection, and almost undetectable level in brain tissues (Fig. 3B). But ACE2 was not increased in intestine post SARS-CoV-2 infection according to our data, which may be contributed by the different infection periods and tissue sampling compared to the previous reports (Lamers et al., 2020; Li et al., 2020; Wrapp et al., 2020; Ziegler et al., 2020). However, several studies have demonstrated the presence of SARS-CoV-2 viral RNA in the brain organoids (Ramani et al., 2020; Zhang et al., 2020a) and brain tissue of COVID-19 patients (Puelles et al., 2020; Song et al., 2021), and the virus could cross the blood-brain barrier (BBB) in a transcellular pathway (Zhang et al., 2021), which together with our findings, support the capability of SARS-CoV-2 infection in the CNS. Consistently, through analyzing the expression of a list of candidate receptors in each tissue post SARS-CoV-2 infection, we identified NRP1 as a receptor of SARS-CoV-2 with a high expression in the infected cerebral cortex. Previous report has shown that NRP1 is ubiquitously expressed in the brain tissues with especially higher level in hippocampal formation (Davies et al., 2020), its expression was elevated in olfactory epithelial cells from human COVID-19 autopsies (Cantuti-Castelvetri et al., 2020), and moreover, NRP1 can mediate entry of nanoparticles coated with SARS-CoV-2 S peptides into the central nervous system of mice (Cantuti-Castelvetri et al., 2020). These collective data support the speculation that NRP1 may serve as the receptor of SARS-CoV-2 in CNS and specifically enhance the brain infection.

A rapid and coordinated immune response during viral infection leads to an enhanced secretion of various inflammatory factors, including interferons, pro-inflammatory cytokines and chemokines, which act as a defense mechanism against the viral infection. Numerous reports suggest that individuals infected with SARS-CoV-2 have dysregulated cytokine production (Harrison et al., 2020). Macrophages infected with SARS-CoV-2 release interferon-stimulated genes (ISGs), which further amplify the expression of pro-inflammatory cytokines and chemokines leading to exacerbated inflammatory responses in COVID-19 patients (Cao, 2021). Recently, neuroinflammation with substantial immune activation in the central nervous system of COVID-19 patients were reported (Schwabenland et al., 2021). Consistently, we detected up-regulation of interferon receptors and ISGs in cortex. In consistent with the findings of SARS-CoV-2 mRNA expression and replication in the brain tissues (Figs. 1 and S1), elevated mRNA and protein levels of macrophage releasing inflammatory cytokines as well as enhanced interstitial infiltration of CD3^+^ T lymphocytes and CD68^+^ macrophages were also observed in infected cerebral cortex (Fig. 4F and 4G). Since influenza RNA virus could induce inflammatory encephalopathy (Wang et al., 2010), we propose that macrophages secrets M1-associated inflammatory cytokines leading to the activated inflammatory responses in neuron and consequently inflammatory encephalopathy post SARS-CoV-2 infection.

In addition, the expression of cytokines, coagulation, angiogenesis and fibrosis related factors was significantly elevated in cerebral cortex post infection, indicating the enhanced immune responses of nervous systems, in particular cerebral cortex post SARS-CoV-2 infection.

Various studies have predicted and reported the adverse neurological and psychiatric outcomes occurring in COVID-19 patients. Moreover, a recent study using a large electronic health records network, provides further evidence for this kind of substantial neurological and psychiatric morbidity in COVID-19 patients (Taquet et al., 2021). In this study, we reveal that the significant transcriptomic changes in cerebral cortex at early stage of SARS-CoV-2 infection illustrate that brain injury might be the prominent feature in SARS-CoV-2 patients. Neuronal receptor pathway might mediate the exposure of brain to the virus in which beta coronaviruses could spread into and directly infect the brain when inhaled as droplets *via* the nasal epithelium (Koyuncu et al., 2013; Dey et al., 2021). Moreover, NRP1 serves as the receptor of SARS-CoV-2 with higher expression in cerebral cortex, which probably contributes to the brain injuries post infection. Besides, recent studies claimed that SARS-CoV-2 infection causes neuroinvasion into cortical neurons with blood-brain barrier leakage (Song et al., 2021; Zhang et al., 2021), and the vascular leakage is linked to immune activation of COVID-19 brains (Schwabenland et al., 2021; Wenzel et al., 2021). The signal transmission among organs relied on systemic blood circulation system post SARS-CoV-2 infection (Fig. S7C), which may further enhance the neuroinflammation. Additionally, inflammatory factors secreted by macrophages and activated T cells cooperatively contribute to the viral encephalitis. Combined these evidences with our findings, we proposed that NRP1 as a receptor enhanced the infection of CNS, then activated the immune response to release inflammatory factors, along with signal transmission among tissues, which further promoted the NRP1 expression and finally led to viral encephalitis in a positive feedback way. These findings extend our current understanding about the drastic transcriptomic changes and further inflammatory responses in brain and cardiovascular systems caused by SARS-CoV-2, which has potential significance in the guidance of clinical therapies of COVID-19 patients.

Following the discovery of SARS-CoV-2, the emergence of multiple variants has been reported, that are more transmissible with a reduced sensitivity to immune mechanisms (Karim and Karim, 2021; Mistry et al., 2021). Subsequently, fast-spreading variants displace local SARS-CoV-2 strain, and were classified as variants of concern (VOCs). These VOCs exhibit enhanced infectivity and transmissibility in human populations, cell cultures, and animal models (Boehm et al., 2021; Cai et al., 2021; Garcia-Beltran et al., 2021; Harvey et al., 2021; Wang et al., 2021a). In addition, their susceptibility to antibody neutralization induced by natural infection or vaccination is partially reduced (Bates et al., 2021; Garcia-Beltran et al., 2021; Harvey et al., 2021). Recent studies have shown that different VOCs exhibit unique intrinsic pathogenic properties within a wide range of tissues and hosts (Stolp et al., 2022). Although these studies suggest that VOCs can transmit more efficiently between hosts and evade humoral immunity, the overall systemic tissue-specific immune responses and molecular mechanisms of tissue-organ damages have not been fully resolved. In our study, we have comprehensively characterized the transcriptional changes of multiple tissues and organs of the infected body, revealed the underlying molecular mechanism of tissue-specific immune responses, and constructed a coordinated regulatory network of multiple tissues and organs at the transcriptome level, which can provide theoretical foundation for clinical personalized treatment and drug usage.

## Materials and methods

### Animal experiments

Rhesus macaques of 3–4 years old were used in an animal biosafety level 3 (ABSL3) facility using HEPA-filtered isolators under pathogen-free conditions. All procedures in this study involving animals were reviewed and approved by the Institutional Animal Care and Use Committee of the Institute of Laboratory Animal Science, Peking Union Medical College (BLL20001-8). All the experiments were complied with all relevant ethical regulations.

Upon intraperitoneally anesthetized by 2.5% avertin with 0.02 mL/g body weight, the rhesus macaques were inoculated intranasally with SARS-CoV-2 stock virus at a dosage of 10^6^ TCID_50_. The infected animals were continuously observed daily to record body weights, clinical symptoms, response to external stimuli and death. The rhesus macaques were dissected at 7 dpi. Major organs were grossly examined and then fixed in 10% buffered formalin solution, and paraffin sections (3–4 µm in thickness) were prepared routinely.

### RNAscope *in situ* hybridization (ISH)

We used RNAscope Multiplex Fluorescent Assay v2 (Advanced Cell Diagnostics, ACD, Cat No.323100) to detect SARS-CoV-2 RNA in formalin fixed and paraffin embedded (FFPE) tissues. RNAscope probe with C1 channel (RNAscope® Probe-V-nCoV2019-S, ACD, Cat No. 848561) is for targeting SARS-CoV-2 positive-sense (genomic) RNA and probe with C2 channel (RNAscope® Probe-V-nCoV2019-S-sense, ACD, Cat No. 845701) is for targeting SARS-CoV-2 negative-sense (a replicative intermediate indicating viral replication) RNA. Briefly, FFPE slides were pretreated by baking, de-paraffinizing, H_2_O_2_-blocking and digestion with Protease Plus. Then the sections were exposed to target probes and incubated at 40 °C in a hybridization oven for 2 h. RNAscope detection was performed by successive incubation at 40 °C with Opal-520 (A-520, FITC, 1:1,500) and Opal-570 (A-570, Cy3, 1:1,500) for 30 min each. Nuclei were counterstained with DAPI and mounted with ProLong Glass Antifade Mountant (Invitrogen, P10144) for TissueGnostics imaging analysis.

### Droplet digital PCR

Droplet digital PCR (ddPCR) was performed *via* the COVID-19 digital PCR detection kit (TargetingOne, Beijing, China), which allows the detection of the *ORF1ab* gene, *N* gene, and a positive reference gene. The detection limit is 100 copies/mL (Table S1).

### Immunohistopathology

Hematoxylin and eosin (HE) and immunohistochemistry (IHC) staining were performed according to standard protocols. The antibodies used in this study are listed in Table S12. Images of HE and IHC staining were recorded using Panoramic MIDI II digital slide scanner (3D Histech).

### RNA purification and RT-qPCR

Total RNAs were extracted from frozen tissues of rhesus macaques using TRI-Reagent (Sigma, T9424) and then subjected to reverse transcription (TOYOBO, FSQ-301). Quantitative real-time PCR was performed by using SYBR qPCR mix (TOYOBO, QPS-201). *GAPDH* was used as an internal control. The primers used in this study are listed in Table S13.

### RNA extraction and rRNA depletion

Total RNAs were extracted from rhesus macaques with TRIzol (ThermoFisher, 15596018), and then subjected to DNase treatment (ThermoFisher, AM2238) and rRNA depletion with the Ribo-off rRNA Depletion Kit (Human/Mouse/Rat) (Vazyme, N406-02) following the manufacturer’s instructions.

### RNA-seq

For each sample, the obtained ribominus RNA was used to construct libraries with the VAHTS® Universal V6 RNA-seq Library Prep Kit (Vazyme, NR604) following the manufacturer’s instructions. Sequencing was performed on the Illumina HiSeq X-Ten sequencing system with paired end 150 bp read length.

### Data processing and analysis

The quality of raw sequencing reads was checked using FastQC (http://www.bioinformatics.babraham.ac.uk/projects/fastqc/), and then adapters were trimmed by Cutadapt (Martin, 2011) (version 1.13). We used Trimmomatic (Bolger et al., 2014) (version 0.36) to filter the reads shorter than 35 nt or containing ambiguous nucleotides. After quality control, the clean reads were aligned to the *Macaca mulatta* (rhesus macaque) genome from Ensembl (Yates et al., 2020) (release 100) using HISAT2 (Kim et al., 2015) (version 2.0.5) with default parameters. Samtools (Li et al., 2009) was used to filter and sort the mapped reads, then featureCounts program (Liao et al., 2014) was applied to summarize the gene counts. Reads per kilobase of exon model per million mapped reads (RPKM) was used to evaluate the genes expression in each sample. Clustering of organs in control group was performed using scaled RPKM by K-means algorithm (Demidenko, 2018) with 10,000 iterations. DEGs between infected and control were identified by edgeR (Robinson et al., 2010) in R program using the threshold ∣log_2_(fold change)∣ ≥ 1 and *P* value < 0.05.

For the functional annotation of gene sets, ClueGO (Bindea et al., 2009) of Cytoscape (Shannon et al., 2003) and DAVID (Huang da et al., 2009a, b) were applied to annotate Gene Ontology Consortium (Ashburner et al., 2000) biological process terms with threshold *P* value < 0.05 and GO term fusion in ClueGO. The reads counts were normalized using bamCoverage of BEDtools (Quinlan and Hall, 2010) v2.26.0 and displayed in the genome by Integrative Genomics Viewer (IGV) (Thorvaldsdottir et al., 2013). UpSetR (Conway et al., 2017) was used to display the overlapped genes in multiple organs.

To predict composition of immune cells in multiple organs, gene expression of RPKM in each sample as input processed using CIBERSORT algorithm (Newman et al., 2015). To protein-protein interaction (PPI) network of fibrosis markers were built using STRING v11.0 (Szklarczyk et al., 2019) using multiple proteins with default parameters. TFs and targets pairs of human were collected from TRRUST version 2 (Han et al., 2018), and ligand-receptor pairs of human were used to build tissues communication from CellTalkDB database (Shao et al., 2020). The polar heatmap of dysregulated TFs was generated by TBtools (Chen et al., 2020).

### Statistics and reproducibility

Statically significant differences between infected and control group were evaluated by Student’s two-side unpaired *t*-test and wilcox test. Statistical significances are presented in the figures as single asterisk (*) when *P* value < 0.05.

## Supplementary Information

Below is the link to the electronic supplementary material.

Supplementary file1 (PDF 2241 kb)

Supplementary file2 (XLSX 14 kb)

Supplementary file3 (XLSX 9 kb)

Supplementary file4 (XLSX 14 kb)

Supplementary file5 (XLSX 20 kb)

Supplementary file6 (XLSX 4837 kb)

Supplementary file7 (XLSX 22345 kb)

Supplementary file8 (XLSX 8755 kb)

Supplementary file9 (XLSX 290 kb)

Supplementary file10 (XLSX 362 kb)

Supplementary file11 (XLSX 544 kb)

Supplementary file12 (XLSX 66 kb)

Supplementary file13 (XLSX 11 kb)

Supplementary file14 (XLSX 9 kb)

Supplementary file15 (XLSX 11 kb)

## References

[CR1] Ackermann M, Verleden SE, Kuehnel M, Haverich A, Welte T, Laenger F, Vanstapel A, Werlein C, Stark H, Tzankov A (2020). Pulmonary vascular endothelialitis, thrombosis, and angiogenesis in COVID-19. N Engl J Med.

[CR2] Ashburner M, Ball CA, Blake JA, Botstein D, Butler H, Cherry JM, Davis AP, Dolinski K, Dwight SS, Eppig JT (2000). Gene ontology: tool for the unification of biology. The Gene Ontology Consortium. Nat Genet.

[CR3] Bates TA, Leier HC, Lyski ZL, McBride SK, Coulter FJ, Weinstein JB, Goodman JR, Lu Z, Siegel SAR, Sullivan P (2021). Neutralization of SARS-CoV-2 variants by convalescent and BNT162b2 vaccinated serum. Nat Commun.

[CR4] Bian X-W, Team tC-P (2020). Autopsy of COVID-19 victims in China. Natl Sci Rev.

[CR5] Bindea G, Mlecnik B, Hackl H, Charoentong P, Tosolini M, Kirilovsky A, Fridman WH, Pages F, Trajanoski Z, Galon J (2009). ClueGO: a cytoscape plug-in to decipher functionally grouped gene ontology and pathway annotation networks. Bioinformatics.

[CR6] Boehm E, Kronig I, Neher RA, Eckerle I, Vetter P, Kaiser L, Centre G, for Emerging Viral, D. (2021). Novel SARS-CoV-2 variants: the pandemics within the pandemic. Clin Microbiol Infect.

[CR7] Bolger AM, Lohse M, Usadel B (2014). Trimmomatic: a flexible trimmer for Illumina sequence data. Bioinformatics.

[CR8] Cai Y, Zhang J, Xiao T, Lavine CL, Rawson S, Peng H, Zhu H, Anand K, Tong P, Gautam A (2021). Structural basis for enhanced infectivity and immune evasion of SARS-CoV-2 variants. Science.

[CR9] Cantuti-Castelvetri L, Ojha R, Pedro LD, Djannatian M, Franz J, Kuivanen S, van der Meer F, Kallio K, Kaya T, Anastasina M (2020). Neuropilin-1 facilitates SARS-CoV-2 cell entry and infectivity. Science.

[CR10] Cao X (2021). ISG15 secretion exacerbates inflammation in SARS-CoV-2 infection. Nat Immunol.

[CR11] Chapin JC, Hajjar KA (2015). Fibrinolysis and the control of blood coagulation. Blood Rev.

[CR12] Chen C, Chen H, Zhang Y, Thomas HR, Frank MH, He Y, Xia R (2020). TBtools: an integrative toolkit developed for interactive analyses of big biological data. Mol Plant.

[CR13] Chen L, Marishta A, Ellison CE, Verzi MP (2021). Identification of transcription factors regulating SARS-CoV-2 entry genes in the intestine. Cell Mol Gastroenterol Hepatol.

[CR14] Conway JR, Lex A, Gehlenborg N (2017). UpSetR: an R package for the visualization of intersecting sets and their properties. Bioinformatics.

[CR31] da Huang W, Sherman BT, Lempicki RA (2009). Bioinformatics enrichment tools: paths toward the comprehensive functional analysis of large gene lists. Nucleic Acids Res.

[CR32] da Huang W, Sherman BT, Lempicki RA (2009). Systematic and integrative analysis of large gene lists using DAVID bioinformatics resources. Nat Protoc.

[CR15] Daly JL, Simonetti B, Klein K, Chen KE, Williamson MK, Anton-Plagaro C, Shoemark DK, Simon-Gracia L, Bauer M, Hollandi R (2020). Neuropilin-1 is a host factor for SARS-CoV-2 infection. Science.

[CR16] Davies J, Randeva HS, Chatha K, Hall M, Spandidos DA, Karteris E, Kyrou I (2020). Neuropilin1 as a new potential SARS-CoV-2 infection mediator implicated in the neurologic features and central nervous system involvement of COVID19. Mol Med Rep.

[CR17] Demidenko E (2018). The next-generation K-means algorithm. Stat Anal Data Min.

[CR18] Deng W, Bao L, Liu J, Xiao C, Liu J, Xue J, Lv Q, Qi F, Gao H, Yu P (2020). Primary exposure to SARS-CoV-2 protects against reinfection in rhesus macaques. Science.

[CR19] Dey J, Alam MT, Chandra S, Gupta J, Ray U, Srivastava AK, Tripathi PP (2021). Neuroinvasion of SARS-CoV-2 may play a role in the breakdown of the respiratory center of the brain. J Med Virol.

[CR20] Fainardi V, Longo F, Chetta A, Esposito S, Pisi G (2020). SARS-CoV-2 infection in patients with cystic fibrosis. An overview. Acta Biomed.

[CR21] Gao Q, Bao L, Mao H, Wang L, Xu K, Yang M, Li Y, Zhu L, Wang N, Lv Z (2020). Development of an inactivated vaccine candidate for SARS-CoV-2. Science.

[CR22] Garcia-Beltran WF, Lam EC, St Denis K, Nitido AD, Garcia ZH, Hauser BM, Feldman J, Pavlovic MN, Gregory DJ, Poznansky MC (2021). Multiple SARS-CoV-2 variants escape neutralization by vaccine-induced humoral immunity. Cell.

[CR23] Giordo R, Paliogiannis P, Mangoni AA, Pintus G (2021). SARS-CoV-2 and endothelial cell interaction in COVID-19: molecular perspectives. Vasc Biol.

[CR24] Gudowska-Sawczuk M, Mroczko B (2021). The role of Neuropilin-1 (NRP-1) in SARS-CoV-2 infection: review. J Clin Med.

[CR25] Gupta A, Madhavan MV, Sehgal K, Nair N, Mahajan S, Sehrawat TS, Bikdeli B, Ahluwalia N, Ausiello JC, Wan EY (2020). Extrapulmonary manifestations of COVID-19. Nat Med.

[CR26] Han H, Cho JW, Lee S, Yun A, Kim H, Bae D, Yang S, Kim CY, Lee M, Kim E (2018). TRRUST v2: an expanded reference database of human and mouse transcriptional regulatory interactions. Nucleic Acids Res.

[CR27] Han H, Yang L, Liu R, Liu F, Wu KL, Li J, Liu XH, Zhu CL (2020). Prominent changes in blood coagulation of patients with SARS-CoV-2 infection. Clin Chem Lab Med.

[CR28] Han X, Zhou Z, Fei L, Sun H, Wang R, Chen Y, Chen H, Wang J, Tang H, Ge W (2020). Construction of a human cell landscape at single-cell level. Nature.

[CR29] Harrison AG, Lin T, Wang P (2020). Mechanisms of SARS-CoV-2 transmission and pathogenesis. Trends Immunol.

[CR30] Harvey WT, Carabelli AM, Jackson B, Gupta RK, Thomson EC, Harrison EM, Ludden C, Reeve R, Rambaut A, Consortium C-GU (2021). SARS-CoV-2 variants, spike mutations and immune escape. Nat Rev Microbiol.

[CR33] Karim SSA, Karim QA (2021). Omicron SARS-CoV-2 variant: a new chapter in the COVID-19 pandemic. Lancet.

[CR35] Kim MS, Pinto SM, Getnet D, Nirujogi RS, Manda SS, Chaerkady R, Madugundu AK, Kelkar DS, Isserlin R, Jain S (2014). A draft map of the human proteome. Nature.

[CR34] Kim D, Langmead B, Salzberg SL (2015). HISAT: a fast spliced aligner with low memory requirements. Nat Methods.

[CR36] Koyuncu OO, Hogue IB, Enquist LW (2013). Virus infections in the nervous system. Cell Host Microbe.

[CR37] Kudose S, Batal I, Santoriello D, Xu K, Barasch J, Peleg Y, Canetta P, Ratner LE, Marasa M, Gharavi AG (2020). Kidney biopsy findings in patients with COVID-19. J Am Soc Nephrol.

[CR38] Lamers MM, Beumer J, van der Vaart J, Knoops K, Puschhof J, Breugem TI, Ravelli RBG, Paul van Schayck J, Mykytyn AZ, Duimel HQ (2020). SARS-CoV-2 productively infects human gut enterocytes. Science.

[CR39] Li H, Handsaker B, Wysoker A, Fennell T, Ruan J, Homer N, Marth G, Abecasis G, Durbin R, Genome Project Data Processing S (2009). The sequence alignment/map format and SAMtools. Bioinformatics.

[CR40] Li MY, Li L, Zhang Y, Wang XS (2020). Expression of the SARS-CoV-2 cell receptor gene ACE2 in a wide variety of human tissues. Infect Dis Poverty.

[CR41] Liao Y, Smyth GK, Shi W (2014). featureCounts: an efficient general purpose program for assigning sequence reads to genomic features. Bioinformatics.

[CR42] Martin M (2011). Cutadapt removes adapter sequences from high-throughput sequencing reads. Embnet J.

[CR43] Mistry P, Barmania F, Mellet J, Peta K, Strydom A, Viljoen IM, James W, Gordon S, Pepper MS (2021). SARS-CoV-2 variants, vaccines, and host immunity. Front Immunol.

[CR44] Mostafavi S, Yoshida H, Moodley D, LeBoite H, Rothamel K, Raj T, Ye CJ, Chevrier N, Zhang SY, Feng T (2016). Parsing the interferon transcriptional network and its disease associations. Cell.

[CR45] Muszbek L, Bereczky Z, Bagoly Z, Komaromi I, Katona E (2011). Factor XIII: a coagulation factor with multiple plasmatic and cellular functions. Physiol Rev.

[CR46] Newman AM, Liu CL, Green MR, Gentles AJ, Feng W, Xu Y, Hoang CD, Diehn M, Alizadeh AA (2015). Robust enumeration of cell subsets from tissue expression profiles. Nat Methods.

[CR47] Nie X, Qian L, Sun R, Huang B, Dong X, Xiao Q, Zhang Q, Lu T, Yue L, Chen S (2021). Multi-organ proteomic landscape of COVID-19 autopsies. Cell.

[CR48] Palta S, Saroa R, Palta A (2014). Overview of the coagulation system. Indian J Anaesth.

[CR49] Peiris JS, Chu CM, Cheng VC, Chan KS, Hung IF, Poon LL, Law KI, Tang BS, Hon TY, Chan CS (2003). Clinical progression and viral load in a community outbreak of coronavirus-associated SARS pneumonia: a prospective study. Lancet.

[CR50] Peyvandi F, Garagiola I, Baronciani L (2011). Role of von Willebrand factor in the haemostasis. Blood Transfus.

[CR51] Puelles VG, Lutgehetmann M, Lindenmeyer MT, Sperhake JP, Wong MN, Allweiss L, Chilla S, Heinemann A, Wanner N, Liu S (2020). Multiorgan and renal tropism of SARS-CoV-2. N Engl J Med.

[CR52] Quinlan AR, Hall IM (2010). BEDTools: a flexible suite of utilities for comparing genomic features. Bioinformatics.

[CR53] Ramani A, Muller L, Ostermann PN, Gabriel E, Abida-Islam P, Muller-Schiffmann A, Mariappan A, Goureau O, Gruell H, Walker A (2020). SARS-CoV-2 targets neurons of 3D human brain organoids. EMBO J.

[CR54] Robinson MD, McCarthy DJ, Smyth GK (2010). edgeR: a bioconductor package for differential expression analysis of digital gene expression data. Bioinformatics.

[CR55] Ronco C, Reis T, Husain-Syed F (2020). Management of acute kidney injury in patients with COVID-19. Lancet Respir Med.

[CR56] Ruan Q, Yang K, Wang W, Jiang L, Song J (2020). Clinical predictors of mortality due to COVID-19 based on an analysis of data of 150 patients from Wuhan, China. Intensive Care Med.

[CR57] Sa Ribero M, Jouvenet N, Dreux M, Nisole S (2020). Interplay between SARS-CoV-2 and the type I interferon response. PLoS Pathog.

[CR58] Schreiber RD, Hicks LJ, Celada A, Buchmeier NA, Gray PW (1985). Monoclonal antibodies to murine gamma-interferon which differentially modulate macrophage activation and antiviral activity. J Immunol.

[CR59] Schwabenland M, Salie H, Tanevski J, Killmer S, Lago MS, Schlaak AE, Mayer L, Matschke J, Puschel K, Fitzek A (2021). Deep spatial profiling of human COVID-19 brains reveals neuroinflammation with distinct microanatomical microglia-T-cell interactions. Immunity.

[CR60] Shannon P, Markiel A, Ozier O, Baliga NS, Wang JT, Ramage D, Amin N, Schwikowski B, Ideker T (2003). Cytoscape: a software environment for integrated models of biomolecular interaction networks. Genome Res.

[CR61] Shao X, Liao J, Li C, Lu X, Cheng J, Fan X (2020). Cell TalkDB: a manually curated database of ligand-receptor interactions in humans and mice. Brief Bioinform.

[CR62] Sica A, Mantovani A (2012). Macrophage plasticity and polarization: in vivo veritas. J Clin Invest.

[CR63] Song E, Zhang C, Israelow B, Lu-Culligan A, Prado AV, Skriabine S, Lu P, Weizman OE, Liu F, Dai Y (2021). Neuroinvasion of SARS-CoV-2 in human and mouse brain. J Exp Med.

[CR64] Stolp B, Stern M, Ambiel I, Hofmann K, Morath K, Gallucci L, Cortese M, Bartenschlager R, Ruggieri A, Graw F (2022). SARS-CoV-2 variants of concern display enhanced intrinsic pathogenic properties and expanded organ tropism in mouse models. Cell Rep.

[CR65] Subramanian A, Tamayo P, Mootha VK, Mukherjee S, Ebert BL, Gillette MA, Paulovich A, Pomeroy SL, Golub TR, Lander ES (2005). Gene set enrichment analysis: a knowledge-based approach for interpreting genome-wide expression profiles. Proc Natl Acad Sci USA.

[CR66] Szklarczyk D, Gable AL, Lyon D, Junge A, Wyder S, Huerta-Cepas J, Simonovic M, Doncheva NT, Morris JH, Bork P (2019). STRING v11: protein-protein association networks with increased coverage, supporting functional discovery in genome-wide experimental datasets. Nucleic Acids Res.

[CR67] Tang X, Yang M, Duan Z, Liao Z, Liu L, Cheng R, Fang M, Wang G, Liu H, Xu J, et al (2020) Transferrin receptor is another receptor for SARS-CoV-2 entry. bioRxiv

[CR68] Taquet M, Geddes JR, Husain M, Luciano S, Harrison PJ (2021). 6-month neurological and psychiatric outcomes in 236379 survivors of COVID-19: a retrospective cohort study using electronic health records. Lancet Psychiatry.

[CR69] Thorvaldsdottir H, Robinson JT, Mesirov JP (2013). Integrative Genomics Viewer (IGV): high-performance genomics data visualization and exploration. Brief Bioinform.

[CR70] Tian S, Xiong Y, Liu H, Niu L, Guo J, Liao M, Xiao SY (2020). Pathological study of the 2019 novel coronavirus disease (COVID-19) through postmortem core biopsies. Mod Pathol.

[CR71] Tipnis SR, Hooper NM, Hyde R, Karran E, Christie G, Turner AJ (2000). A human homolog of angiotensin-converting enzyme. Cloning and functional expression as a captopril-insensitive carboxypeptidase. J Biol Chem.

[CR72] Varga Z, Flammer AJ, Steiger P, Haberecker M, Andermatt R, Zinkernagel AS, Mehra MR, Schuepbach RA, Ruschitzka F, Moch H (2020). Endothelial cell infection and endotheliitis in COVID-19. Lancet.

[CR73] Vial C, Calderon JF, Klein AD (2021). NPC1 as a modulator of disease severity and viral entry of SARS-CoV- 2. Curr Mol Med.

[CR74] von Weyhern CH, Kaufmann I, Neff F, Kremer M (2020). Early evidence of pronounced brain involvement in fatal COVID-19 outcomes. The Lancet.

[CR76] Wang GF, Li W, Li K (2010). Acute encephalopathy and encephalitis caused by influenza virus infection. Curr Opin Neurol.

[CR75] Wang D, Hu B, Hu C, Zhu F, Liu X, Zhang J, Wang B, Xiang H, Cheng Z, Xiong Y (2020). Clinical characteristics of 138 hospitalized patients with 2019 novel coronavirus-infected pneumonia in Wuhan, China. JAMA.

[CR77] Wang R, Zhang Q, Ge J, Ren W, Zhang R, Lan J, Ju B, Su B, Yu F, Chen P (2021). Analysis of SARS-CoV-2 variant mutations reveals neutralization escape mechanisms and the ability to use ACE2 receptors from additional species. Immunity.

[CR78] Wang S, Qiu Z, Hou Y, Deng X, Xu W, Zheng T, Wu P, Xie S, Bian W, Zhang C (2021). AXL is a candidate receptor for SARS-CoV-2 that promotes infection of pulmonary and bronchial epithelial cells. Cell Res.

[CR79] Wenzel J, Lampe J, Muller-Fielitz H, Schuster R, Zille M, Muller K, Krohn M, Korbelin J, Zhang L, Ozorhan U (2021). The SARS-CoV-2 main protease M^pro^ causes microvascular brain pathology by cleaving NEMO in brain endothelial cells. Nat Neurosci.

[CR80] Wichmann D (2020). Autopsy findings and venous thromboembolism in patients with COVID-19. Ann Intern Med.

[CR81] Wichmann D, Sperhake JP, Lutgehetmann M, Steurer S, Edler C, Heinemann A, Heinrich F, Mushumba H, Kniep I, Schroder AS (2020). Autopsy findings and venous thromboembolism in patients with COVID-19: a prospective cohort study. Ann Intern Med.

[CR82] Wrapp D, Wang N, Corbett KS, Goldsmith JA, Hsieh CL, Abiona O, Graham BS, McLellan JS (2020). Cryo-EM structure of the 2019-nCoV spike in the prefusion conformation. Science.

[CR83] Yang M, Chen S, Huang B, Zhong JM, Su H, Chen YJ, Cao Q, Ma L, He J, Li XF (2020). Pathological findings in the testes of COVID-19 patients: clinical implications. Eur Urol Focus.

[CR84] Yates AD, Achuthan P, Akanni W, Allen J, Allen J, Alvarez-Jarreta J, Amode MR, Armean IM, Azov AG, Bennett R (2020). Ensembl 2020. Nucleic Acids Res.

[CR85] Yu F, Yan L, Wang N, Yang S, Wang L, Tang Y, Gao G, Wang S, Ma C, Xie R (2020). Quantitative detection and viral load analysis of SARS-CoV-2 in infected patients. Clin Infect Dis.

[CR86] Yu P, Qi F, Xu Y, Li F, Liu P, Liu J, Bao L, Deng W, Gao H, Xiang Z (2020). Age-related rhesus macaque models of COVID-19. Animal Model Exp Med.

[CR87] Zhang BZ, Chu H, Han S, Shuai H, Deng J, Hu YF, Gong HR, Lee AC, Zou Z, Yau T (2020). SARS-CoV-2 infects human neural progenitor cells and brain organoids. Cell Res.

[CR88] Zhang C, Shi L, Wang F-S (2020). Liver injury in COVID-19: management and challenges. Lancet Gastroenterol Hepatol.

[CR89] Zhang L, Zhou L, Bao L, Liu J, Zhu H, Lv Q, Liu R, Chen W, Tong W, Wei Q (2021). SARS-CoV-2 crosses the blood-brain barrier accompanied with basement membrane disruption without tight junctions alteration. Signal Transduct Target Ther.

[CR90] Zheng YY, Ma YT, Zhang JY, Xie X (2020). COVID-19 and the cardiovascular system. Nat Rev Cardiol.

[CR91] Zhou F, Yu T, Du R, Fan G, Liu Y, Liu Z, Xiang J, Wang Y, Song B, Gu X (2020). Clinical course and risk factors for mortality of adult inpatients with COVID-19 in Wuhan, China: a retrospective cohort study. Lancet.

[CR92] Zhu N, Zhang D, Wang W, Li X, Yang B, Song J, Zhao X, Huang B, Shi W, Lu R (2020). A novel coronavirus from patients with pneumonia in China, 2019. N Engl J Med.

[CR93] Ziegler CGK, Allon SJ, Nyquist SK, Mbano IM, Miao VN, Tzouanas CN, Cao Y, Yousif AS, Bals J, Hauser BM (2020). SARS-CoV-2 receptor ACE2 is an interferon-stimulated gene in human airway epithelial cells and is detected in specific cell subsets across tissues. Cell.

