# Supplementary material for: Differential transcriptomic landscapes of multiple organs from SARS-CoV-2 early infected rhesus macaques
Source: Protein Cell. 2022 Apr 4;13(12):920–39. doi: 10.1007/s13238-022-00915-5 (PMC8978510; doi:10.1007/s13238-022-00915-5)
Supplement: Supplementary file 1 — Supplementary file1 (PDF 2241 kb) [file 13238_2022_915_MOESM1_ESM.pdf]

Supplementary Materials

Differential transcriptomic landscapes of multiple organs from SARS-CoV-2 early infected rhesus macaques

Chun-Chun Gao, Man Li, Wei Deng, Yu-Sheng Chen, Yong-Qiao Sun, Tingfu Du, Qian-Lan Liu, Wen-Jie Li, Bing Zhang, Lihong Sun, Si-Meng Liu, Fengli Li, Feifei Qi, Yajin Qu, Xin-Yang Ge, Jiangning Liu, Peng Wang, Yamei Niu, Zhiyong Liang, Yong-Liang Zhao, Bo Huang, Xiao-Zhong Peng, Xiaozhong Peng, Ying Yang, Chuan Qin, Wei-Min Tong, Yun-Gui Yang

Supplementary Legends

Supplementary Fig. S1 *In situ* hybridization of SARS-CoV-2 in liver, kidney, micro-vessel, testis and colon of infected rhesus macaques.

A. RNAscope images of SARS-CoV-2 expression (Green, FITC-labelled) and replication (Red, Cy3-labelled) in testis. Hematoxylin and Eosin (HE) staining were used to refer the SARS-CoV-2 *in situ* distribution and replication in each tissue. Scale bars, HE 100 μm and ISH 20 μm.

B. RNAscope images of SARS-CoV-2 expression and replication in liver. Legend as in **A**.

C. RNAscope images of SARS-CoV-2 expression and replication in kidney. Legend as in **A**.

D. RNAscope images of SARS-CoV-2 expression and replication in micro-vessel. Legend as in **A**.

E. RNAscope images of SARS-CoV-2 expression and replication in colon. Legend as in **A**.

F. Droplet digital PCR for viral *ORF1ab* and *N* sgRNA quantification in all 14 tissues of three SARS-CoV-2 infected macaques for 7 days. Using a viral load > 10 $\log_{10}$ (copies/ml) as the threshold of positivity-tissue-based PCR. The asterisk and diamond-shaped blocks represented the *ORF1ab* and *N* segments of SARS-CoV-2, respectively.

Supplementary Fig. S2 Transcriptomic characteristics for multiple tissues in rhesus macaque.

A. Violin plot showing expression profiling for specific tissue marker genes of stomach (left), liver (middle) and cerebral cortex (right) in all of 14 tissues.

B. Heatmap showing correlation coefficient for expression profiling between each two tissues. The tissues have been divided into different groups by hierarchical clustering.

C-H. Line plots showing the normalized expression patterns for genes from other 6 clusters, individually, which are determined by K-means clustering analysis. The light lines represent normalized expression value for each gene from different clusters, while the dark lines represent mean of normalized expression values among all genes from each cluster.

I-N. Barplots showing the enriched Gene Ontology (GO) terms for other 6 clusters, individually, which are corresponding to genes in (**C-H**).

Supplementary Fig. S3 Opposite transcriptomic changes from cerebral cortex *versus* cerebellum and right ventricle in rhesus macaques post SARS-CoV-2 infection.

A. Heatmap showing high similarity of infected samples in each tissue. F represents female, M is males and 1 or 2 represents the individuals.

B. The t-distributed Stochastic Neighbor Embedding (t-SNE) visualization of gene expression patterns for different tissues from control and infected rhesus macaques.

C. Bubble chart showing the enrichment of GO terms for significantly down-regulated genes identified in each tissue. Bubble size represents counts of identified down-regulated genes in each term for individual tissue, and *P* values from non-significance to high significance are shown as blue to red.

D. Heatmap showing correlation coefficient for expression profiling between each two samples from cerebral cortex, cerebellum and right ventricle.

E. Heatmap showing the fold changes of dysregulated genes in cerebral cortex, right ventricle or cerebellum of rhesus macaques post infection. The fold change of each gene is then normalized by *Z*-score among three tissues.

F. Venn plots displaying the intersection of dysregulated genes among cerebral cortex, cerebellum and right ventricle. The genes have been divided to two group: (i) up-regulated in cerebral cortex but down-regulated in cerebellum or right ventricle (left); (ii) down-regulated in cerebral cortex but up-regulated in cerebellum or right ventricle (right).

G. Map of enriched GO functional terms for 1709 dysregulated genes in cerebral cortex, right ventricle and cerebellum, which are corresponding to **F**.

H. Map of enriched GO functional terms for 1287 dysregulated genes in cerebral cortex, right ventricle and cerebellum, which are corresponding to **F**.

Supplementary Fig. S4 Opposite gene expression patterns of immune response between cerebral cortex and cerebellum/right ventricle tissues.

A. Genome browser showing the reads abundance along *IFNLR1* in cerebral cortex, cerebellum and right ventricle from control and infected rhesus macaques.

B. Volcano plot showing the difference in all expressed interferon-stimulated genes (ISGs) between control and infected samples in right ventricle (left) and cerebellum (right). Purple and brown points: up-regulated ISGs; green and dark green points: down-regulated ISGs.

C. Intersection analysis of dysregulated ISGs in cerebral cortex, right ventricle and cerebellum of infected rhesus macaques. Only up-regulated ISGs for cerebral cortex are selected, while down-regulated ISGs for cerebellum and right ventricle, respectively.

D. Map of enriched GO functional terms for 112 common dysregulated ISGs in cerebral cortex, right ventricle and cerebellum of infected rhesus macaques (corresponding to **C**). Dot represents enriched GO terms. Size of dot stands for its significance level (*P* value); Line means the shared genes by two terms; Width of lines stands for the counts of shared genes.

E. Heatmap showing the fold changes of M2 macrophage related genes after infection for each of 14 tissues, individually. The fold change of each gene is then normalized by *Z*-score among tissues, while asterisk represents dysregulated genes with statistical significance ($P < 0.05$) identified in each tissue.

F. Pie charts showing the estimated proportions for different types of immune cells in cerebral cortex (top), right ventricle (middle) and cerebellum (bottom) of control (left) and infected (right) samples.

Supplementary Fig. S5 Network of elevated transcription factors and their targets in cerebral cortex, cerebellum and right ventricle post SARS-CoV-2 infection.

A. Polar heatmap showing the fold changes of significantly dysregulated TFs in each tissue from control and infected rhesus macaques. *Z*-score represented the scaled log_2(fold change).

B. Network showing the interactions between significantly elevated TFs and their targets in cerebral cortex post infection based on TF-target pairs using Cytoscape software. Red diamonds were the induced TFs, and circular dots represented their targets. Light purple dots were up-regulated targets in cerebral cortex post infection, light green ones were down-regulated targets and light blue ones were the unchanged targets. The size of TFs represents the number of their interaction pairs, and the size of targets represents their fold change post infection.

C. Network showing the interactions between significantly elevated TFs and their targets in cerebellum post infection based on TF-target pairs using Cytoscape software. Legend as **B**.

D. Network showing the interactions between significantly elevated TFs and their targets in right ventricle post infection based on TF-target pairs using Cytoscape software. Legend as **B**.

Supplementary Fig. S6 Dysregulated angiogenesis and stage-specific fibrosis factors in multiple tissues.

A. The number of significantly dysregulated genes of cytokine, coagulation, angiogenesis and fibrosis across 14 tissues in rhesus macaques post infection.

B. Map of enriched GO functional terms for significantly up-regulated cytokines (corresponding to Fig. 6**A**) in cerebral cortex of infected rhesus macaque.

C. Map of enriched GO functional terms for significantly up-regulated coagulation (corresponding to Fig. 6**B**) in cerebral cortex of infected rhesus macaque.

D. Map of enriched GO functional terms for significantly up-regulated angiogenesis (corresponding to Fig. 6**C**) in cerebral cortex of infected rhesus macaque.

E. Interaction network showing the stage-specific dysregulated fibrotic genes in the other 11 tissues post SARS-CoV-2 infection over four fibrosis stages, including initiation (green), inflammation (light blue), proliferation (orange) and modification (red). The interactions were built based on the STRING database using Cytoscape software. The size of bubbles represents the value of fold change of dysregulated genes from infected rhesus macaques compared to the control one.

Supplementary Fig. S7 Occurrence of encephalitis post SARS-CoV-2 infection.

A. Hematoxylin and eosin (HE) and Immunohistochemical (IHC) staining showing induced expression of VEGFA and TNFRSF1 in infected cerebral cortex.

B. enrichment of GO terms for significantly dysregulated transported ligands from cerebellum and right ventricle to cerebral cortex.

C. Schematic diagram displaying the two potential pathways for cerebral cortex infection. (i) Neuronal receptor pathway: NRP1 served as neuronal receptor of SARS-CoV-2 in nasal epithelium and cerebral cortex, contributing to cerebral cortex infection; (ii) Signal transduction pathway along blood circulation system: secreted neurotransmitter from right ventricle post SARS-CoV-2 infection might induce hormones secretion, which transport *via* the circulatory system until to the cerebral cortex and induce response for virus infection.

Supplementary Table Legends

Supplementary Table S1. Viral RNA copies in the detected organs. Using 10 copy equivalents of viral RNA as the limits of detection for SARS-CoV-2.

Supplementary Table S2. Distribution and expression of SARS-CoV-2 in multiple tissues of rhesus macaque. The cell types in multiple tissues were used to detect the distribution and expression of SARS-CoV-2 in rhesus macaques.

Supplementary Table S3. Sequencing depth and alignment statistics for RNA-seq in multiple tissues samples.

Supplementary Table S4. Expression of tissues marker genes for multiple tissues in rhesus macaque. Tissue marker genes were collected from Human Cell Landscape database, the expression value was the evaluated as reads kilobase per million mapped reads (RPKM) in each tissue.

Supplementary Table S5. Transcriptomic K-means clustering results for multiple tissues of rhesus macaque. K-means clustering was performed to classify genes among 14 tissues by *Z*-score normalized expression. Total 8 clusters were summarized for all tissues in rhesus macaque.

Supplementary Table S6. Expression profiling for multiple tissues of rhesus macaque post SARS-CoV-2 infection. RPKM was used to evaluate genes expression in all tissues of control and infected rhesus macaques.

Supplementary Table 7. Profiling of differentially expressed genes among multiple tissues in rhesus macaque post SARS-CoV-2 infection. Compared infected samples to the control one for each tissue, significantly dysregulated genes with $|\text{fold change}| \geq 2$ and P value < 0.05 in at least one tissue were summarized.

Supplementary Table S8. Changes of IFN-stimulated genes among multiple tissues in rhesus macaque post SARS-CoV-2 infection. Total 572 ISGs were summarized with fold change and *P* value for each tissue from control and infected rhesus macaque.

Supplementary Table S9. Changes of transcription factors among multiple tissues

in rhesus macaque post SARS-CoV-2 infection. Total 745 TFs were summarized with fold change and *P* value for each tissue from control and infected rhesus macaque.

Supplementary Table S10. Changes of receptors, cytokines, coagulation, angiogenesis, and fibrosis factors among multiple tissues in rhesus macaque post SARS-CoV-2 infection. Multiple factors were summarized with fold change and *P* value for each tissue from control and infected rhesus macaque.

Supplementary Table S11. Up-regulated ligand-receptor pairs among multiple organs in rhesus macaque post SARS-CoV-2 infection.

Supplementary Table S12. Antibodies used in this study.

Supplementary Table S13. Primers for RT-qPCR in this study.

Supplementary Table S14. Source data for RT-qPCR.

Figure S1

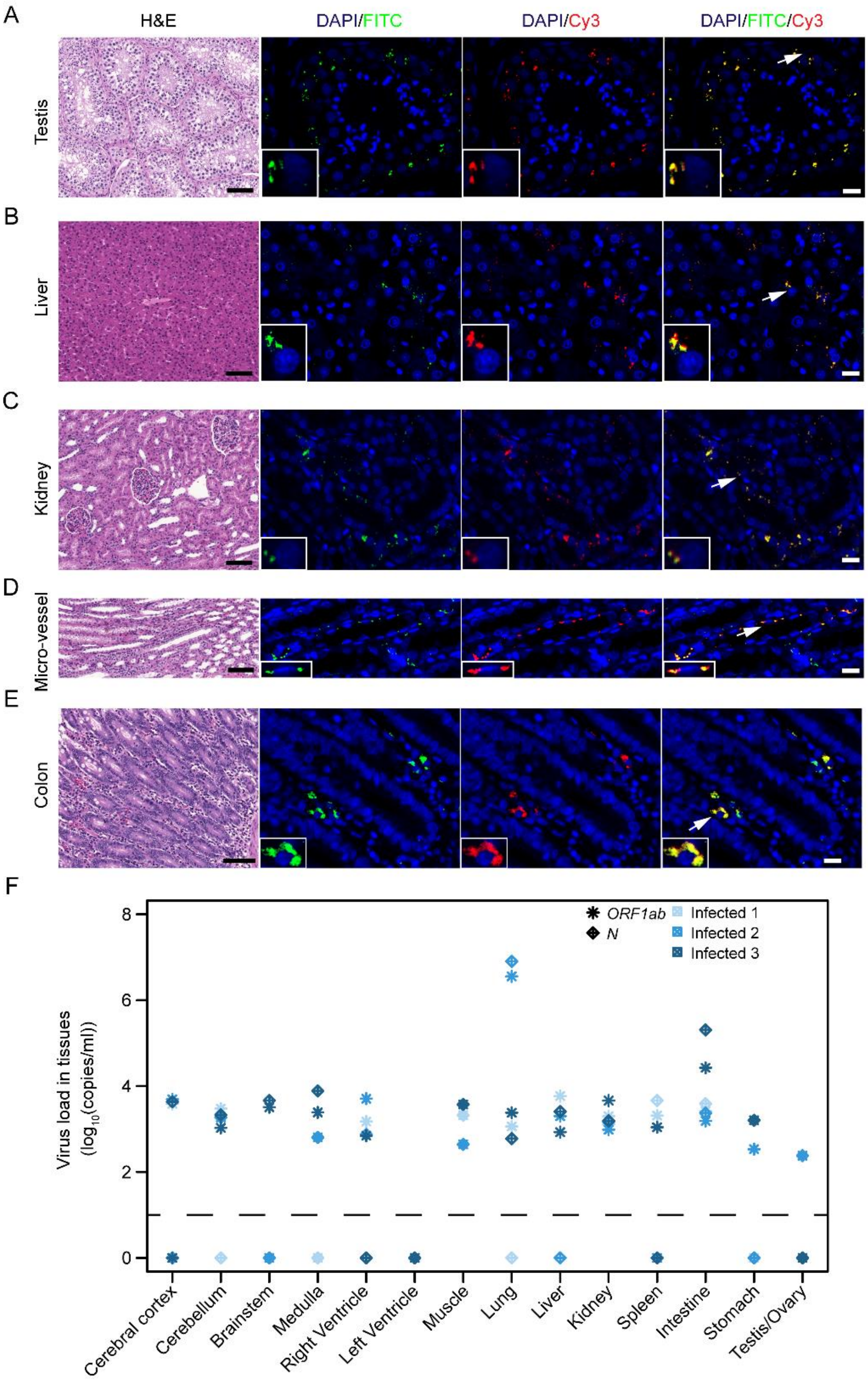
A
H&E
DAPI/FITC
DAPI/Cy3
DAPI/FITC/Cy3
Testis
B
Liver
C
Kidney
D
Micro-vessel
E
Colon
F
ORF1ab
N
Infected 1
Infected 2
Infected 3
Virus load in tissues (log10(copies/ml))
8
6
4
2
0
Cerebral cortex
Cerebellum
Brainstem
Medulla
Right Ventricle
Left Ventricle
Muscle
Lung
Liver
Kidney
Spleen
Intestine
Stomach
Testis/Ovary

Figure S2

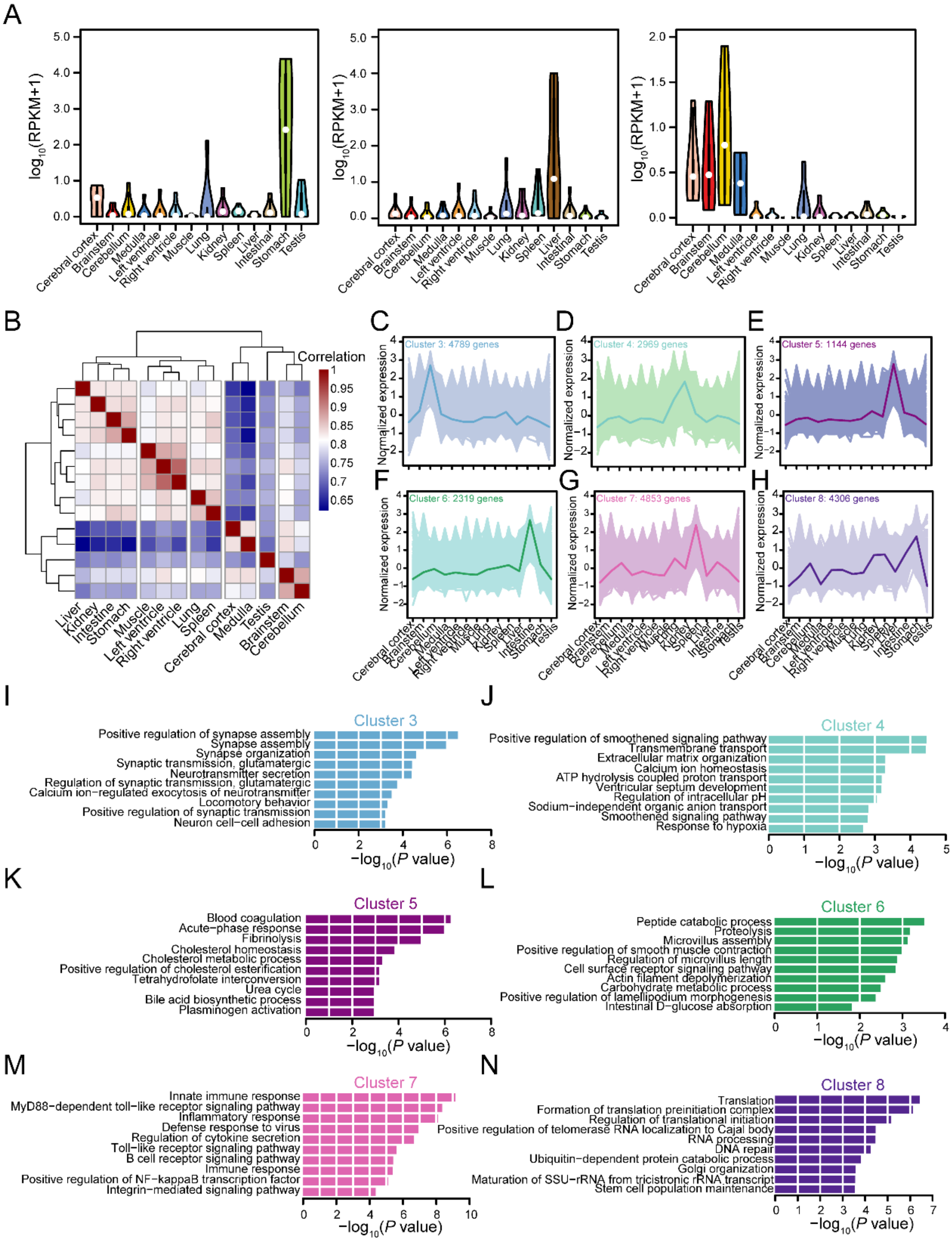

A
B
C
D
E
F
G
H
I
J
K
L
M
N
log10(RPKM+1)
Cerebral cortex
Brainstem
Cerebellum
Medulla
Left ventricle
Right ventricle
Muscle
Lung
Kidney
Spleen
Liver
Intestinal
Stomach
Testis
Correlation
Cluster 3: 4789 genes
Cluster 4: 2969 genes
Cluster 5: 1144 genes
Cluster 6: 2319 genes
Cluster 7: 4853 genes
Cluster 8: 4306 genes
Normalized expression
Cluster 3
Positive regulation of synapse assembly
Synapse assembly
Synapse organization
Synaptic transmission, glutamatergic
Neurotransmitter secretion
Regulation of synaptic transmission, glutamatergic
Calcium ion−regulated exocytosis of neurotransmitter
Locomotory behavior
Positive regulation of synaptic transmission
Neuron cell−cell adhesion
Cluster 4
Positive regulation of smoothened signaling pathway
Transmembrane transport
Extracellular matrix organization
Calcium ion homeostasis
ATP hydrolysis coupled proton transport
Ventricular septum development
Regulation of intracellular pH
Sodium−independent organic anion transport
Smoothened signaling pathway
Response to hypoxia
Cluster 5
Blood coagulation
Acute−phase response
Fibrinolysis
Cholesterol homeostasis
Cholesterol metabolic process
Positive regulation of cholesterol esterification
Tetrahydrofolate interconversion
Urea cycle
Bile acid biosynthetic process
Plasminogen activation
Cluster 6
Peptide catabolic process
Proteolysis
Microvillus assembly
Positive regulation of smooth muscle contraction
Regulation of microvillus length
Cell surface receptor signaling pathway
Actin filament depolymerization
Carbohydrate metabolic process
Positive regulation of lamellipodium morphogenesis
Intestinal D−glucose absorption
Cluster 7
Innate immune response
MyD88−dependent toll−like receptor signaling pathway
Inflammatory response
Defense response to virus
Regulation of cytokine secretion
Toll−like receptor signaling pathway
B cell receptor signaling pathway
Immune response
Positive regulation of NF−kappaB transcription factor
Integrin−mediated signaling pathway
Cluster 8
Translation
Formation of translation preinitiation complex
Regulation of translational initiation
Positive regulation of telomerase RNA localization to Cajal body
RNA processing
DNA repair
Ubiquitin−dependent protein catabolic process
Golgi organization
Maturation of SSU−rRNA from tricistronic rRNA transcript
Stem cell population maintenance
−log10(P value)

Figure S3

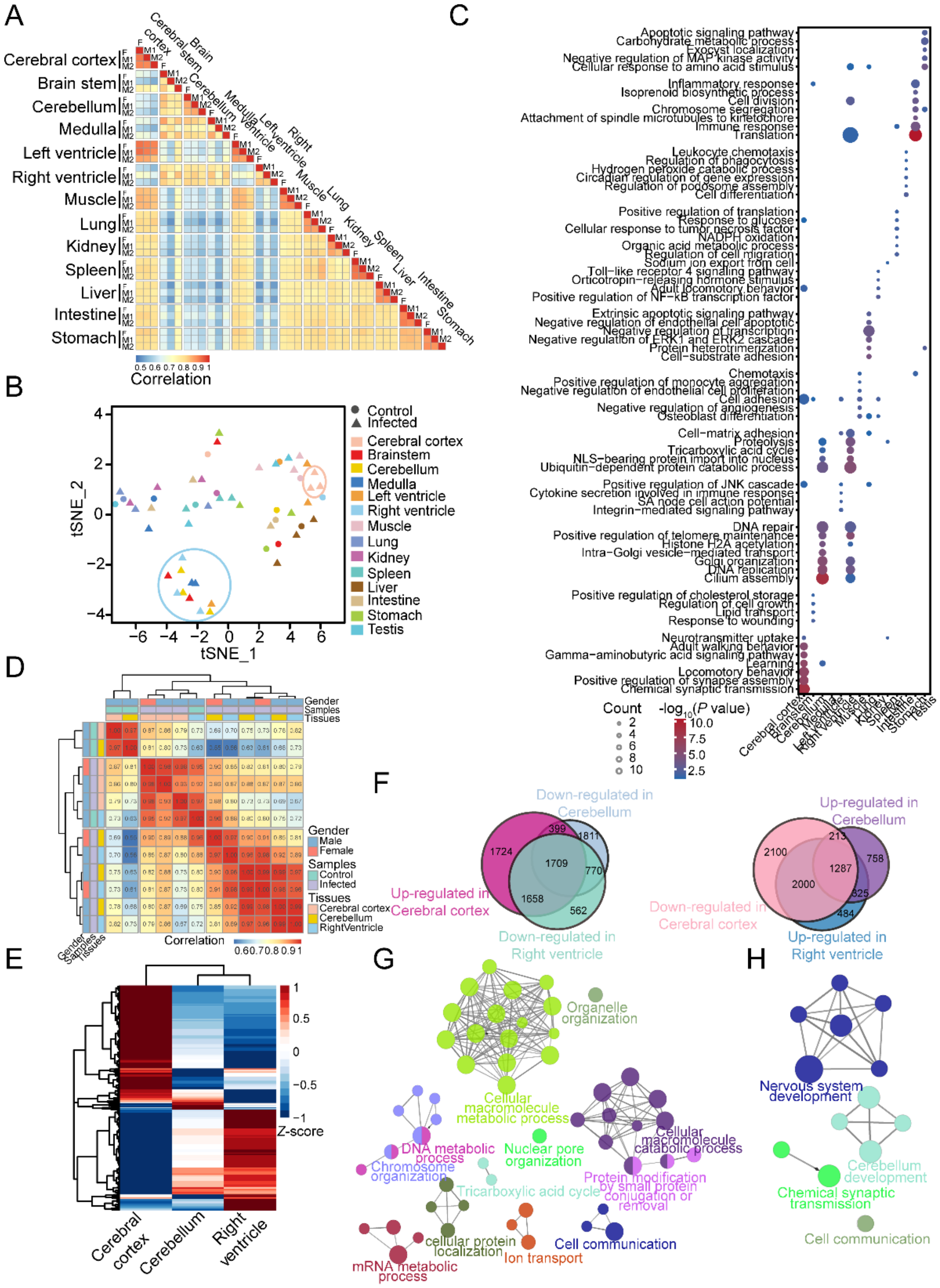
A
Cerebral cortex
Brain stem
Cerebellum
Medulla
Left ventricle
Right ventricle
Muscle
Lung
Kidney
Spleen
Liver
Intestine
Stomach
Correlation
B
Control
Infected
Cerebral cortex
Brainstem
Cerebellum
Medulla
Left ventricle
Right ventricle
Muscle
Lung
Kidney
Spleen
Liver
Intestine
Stomach
Testis
tSNE_1
tSNE_2
C
Apoptotic signaling pathway
Carbohydrate metabolic process
Exocyst localization
Negative regulation of MAP kinase activity
Cellular response to amino acid stimulus
Inflammatory response
Isoprenoid biosynthetic process
Cell division
Chromosome segregation
Attachment of spindle microtubules to kinetochore
Immune response
Translation
Leukocyte chemotaxis
Regulation of phagocytosis
Hydrogen peroxide catabolic process
Circadian regulation of gene expression
Regulation of podosome assembly
Cell differentiation
Positive regulation of translation
Response to glucose
Cellular response to tumor necrosis factor
NADPH oxidation
Organic acid metabolic process
Regulation of cell migration
Sodium ion export from cell
Toll−like receptor 4 signaling pathway
Corticotropin−releasing hormone stimulus
Adult locomotory behavior
Positive regulation of NF−kB transcription factor
Extrinsic apoptotic signaling pathway
Negative regulation of endothelial cell apoptotic
Negative regulation of transcription
Negative regulation of ERK1 and ERK2 cascade
Protein heterotrimerization
Cell−substrate adhesion
Chemotaxis
Positive regulation of monocyte aggregation
Negative regulation of endothelial cell proliferation
Cell adhesion
Negative regulation of angiogenesis
Osteoblast differentiation
Cell−matrix adhesion
Proteolysis
Tricarboxylic acid cycle
NLS−bearing protein import into nucleus
Ubiquitin−dependent protein catabolic process
Positive regulation of JNK cascade
Cytokine secretion involved in immune response
SA node cell action potential
Integrin−mediated signaling pathway
DNA repair
Positive regulation of telomere maintenance
Histone H2A acetylation
Intra−Golgi vesicle−mediated transport
Golgi organization
DNA replication
Cilium assembly
Positive regulation of cholesterol storage
Regulation of cell growth
Lipid transport
Response to wounding
Neurotransmitter uptake
Adult walking behavior
Gamma−aminobutyric acid signaling pathway
Learning
Locomotory behavior
Positive regulation of synapse assembly
Chemical synaptic transmission
Count
-log10(P value)
Cerebral cortex
Brainstem
Cerebellum
Medulla
Left ventricle
Right ventricle
Muscle
Lung
Kidney
Spleen
Liver
Intestine
Stomach
Testis
D
Gender
Samples
Tissues
Male
Female
Control
Infected
Cerebral cortex
Cerebellum
RightVentricle
Correlation
E
Z-score
Cerebral cortex
Cerebellum
Right ventricle
F
Down-regulated in Cerebellum
Up-regulated in Cerebral cortex
Down-regulated in Right ventricle
Up-regulated in Cerebellum
Down-regulated in Cerebral cortex
Up-regulated in Right ventricle
G
Organelle organization
Cellular macromolecule metabolic process
DNA metabolic process
Nuclear pore organization
Cellular macromolecule catabolic process
Chromosome organization
Tricarboxylic acid cycle
Protein modification by small protein conjugation or removal
cellular protein localization
mRNA metabolic process
Ion transport
Cell communication
H
Nervous system development
Cerebellum development
Chemical synaptic transmission
Cell communication

Figure S4

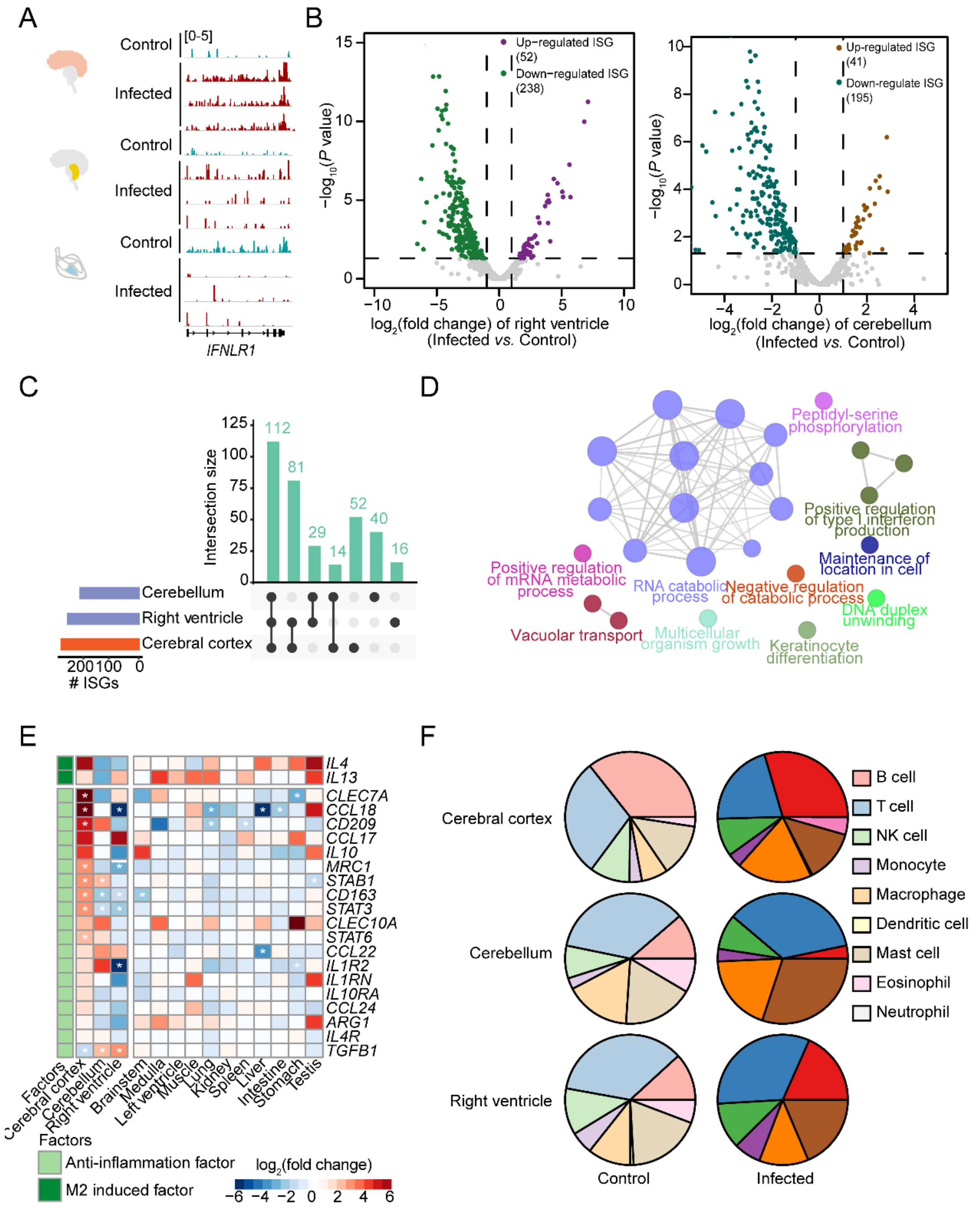

A
Control
Infected
[0-5]
IFNLR1
B
Up−regulated ISG (52)
Down−regulated ISG (238)
−log10(P value)
log2(fold change) of right ventricle (Infected vs. Control)
Up−regulated ISG (41)
Down−regulate ISG (195)
log2(fold change) of cerebellum (Infected vs. Control)
C
Intersection size
112
81
29
14
52
40
16
Cerebellum
Right ventricle
Cerebral cortex
ISGs
D
Peptidyl-serine phosphorylation
Positive regulation of type I interferon production
Maintenance of location in cell
Positive regulation of mRNA metabolic process
RNA catabolic process
Negative regulation of catabolic process
DNA duplex unwinding
Vacuolar transport
Multicellular organism growth
Keratinocyte differentiation
E
IL4
IL13
CLEC7A
CCL18
CD209
CCL17
IL10
MRC1
STAB1
CD163
STAT3
CLEC10A
STAT6
CCL22
IL1R2
IL1RN
IL10RA
CCL24
ARG1
IL4R
TGFB1
Factors
Cerebral cortex
Cerebellum
Right ventricle
Brainstem
Medulla
Left ventricle
Muscle
Lung
Kidney
Spleen
Liver
Intestine
Stomach
Testis
Anti-inflammation factor
M2 induced factor
log2(fold change)
F
Cerebral cortex
Cerebellum
Right ventricle
Control
Infected
B cell
T cell
NK cell
Monocyte
Macrophage
Dendritic cell
Mast cell
Eosinophil
Neutrophil

Figure S5

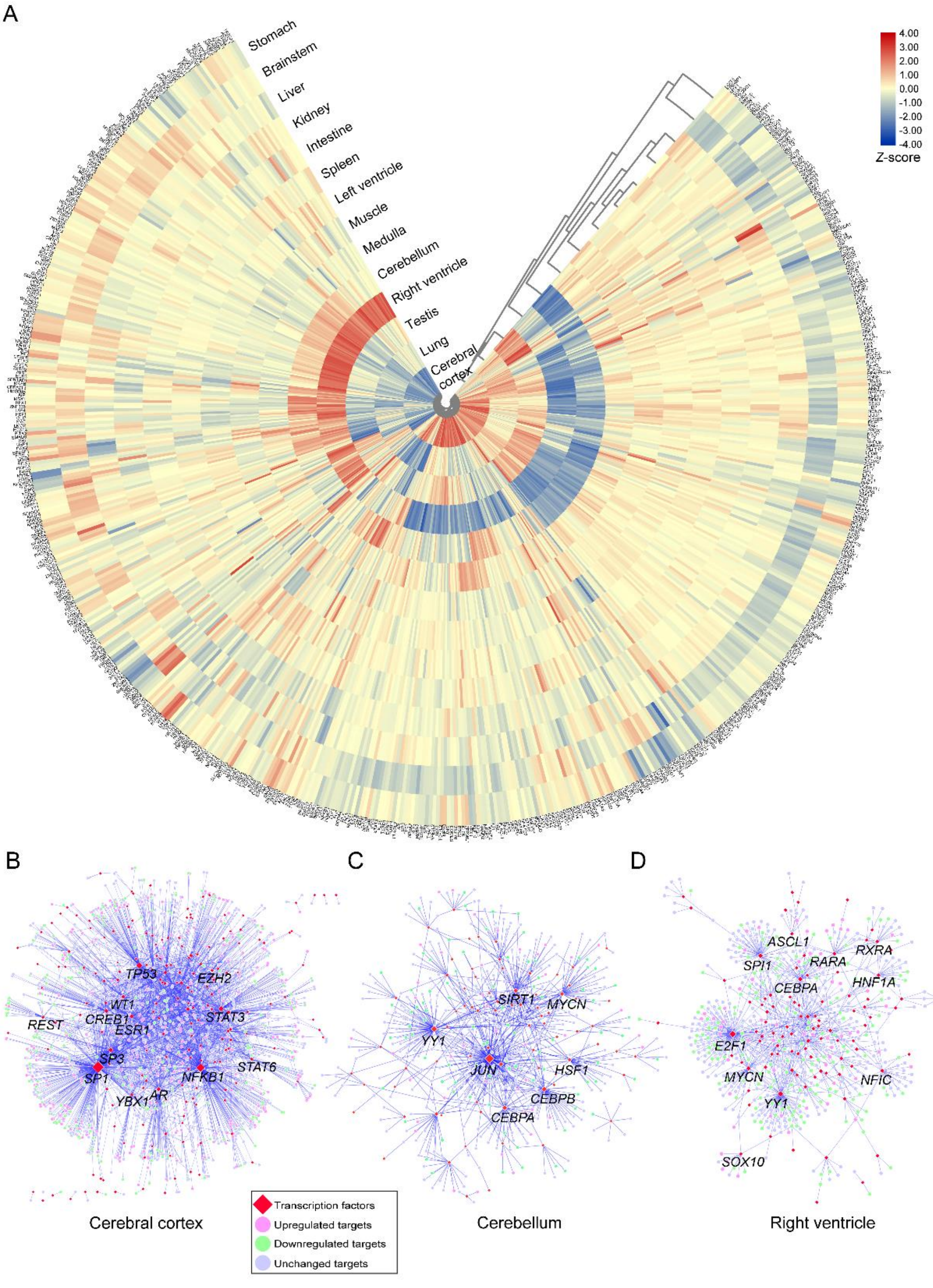
A
Stomach
Brainstem
Liver
Kidney
Intestine
Spleen
Left ventricle
Muscle
Medulla
Cerebellum
Right ventricle
Testis
Lung
Cerebral cortex
4.00
3.00
2.00
1.00
0.00
-1.00
-2.00
-3.00
-4.00
Z-score
B
C
D
TP53
EZH2
WT1
CREB1
ESR1
STAT3
REST
SP3
SP1
NFKB1
STAT6
YBX1
AR
Cerebral cortex
SIRT1
MYCN
YY1
JUN
HSF1
CEBPB
CEBPA
Cerebellum
ASCL1
SPI1
RARA
RXRA
CEBPA
HNF1A
E2F1
MYCN
NFIC
YY1
SOX10
Right ventricle
Transcription factors
Upregulated targets
Downregulated targets
Unchanged targets

Figure S6

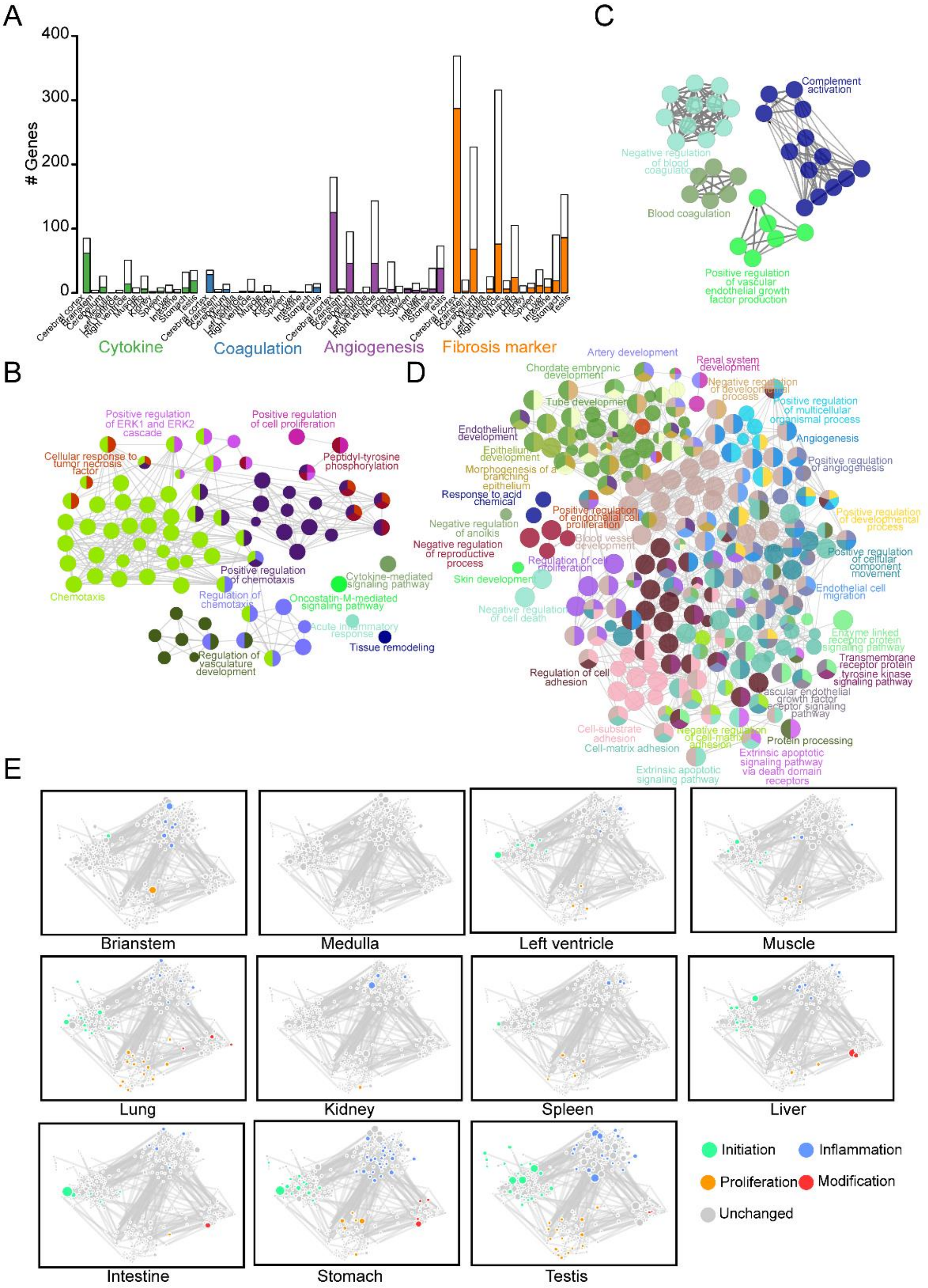
A
Genes
Cytokine
Coagulation
Angiogenesis
Fibrosis marker
B
Positive regulation of ERK1 and ERK2 cascade
Positive regulation of cell proliferation
Cellular response to tumor necrosis factor
Peptidyl-tyrosine phosphorylation
Chemotaxis
Positive regulation of chemotaxis
Regulation of chemotaxis
Cytokine-mediated signaling pathway
Oncostatin-M-mediated signaling pathway
Acute inflammatory response
Tissue remodeling
Regulation of vasculature development
C
Complement activation
Negative regulation of blood coagulation
Blood coagulation
Positive regulation of vascular endothelial growth factor production
D
Artery development
Renal system development
Chordate embryonic development
Tube development
Angiogenesis
Positive regulation of angiogenesis
Regulation of cell adhesion
Cell-matrix adhesion
Protein processing
E
Brianstem
Medulla
Left ventricle
Muscle
Lung
Kidney
Spleen
Liver
Intestine
Stomach
Testis
Initiation
Inflammation
Proliferation
Modification
Unchanged

Figure S7

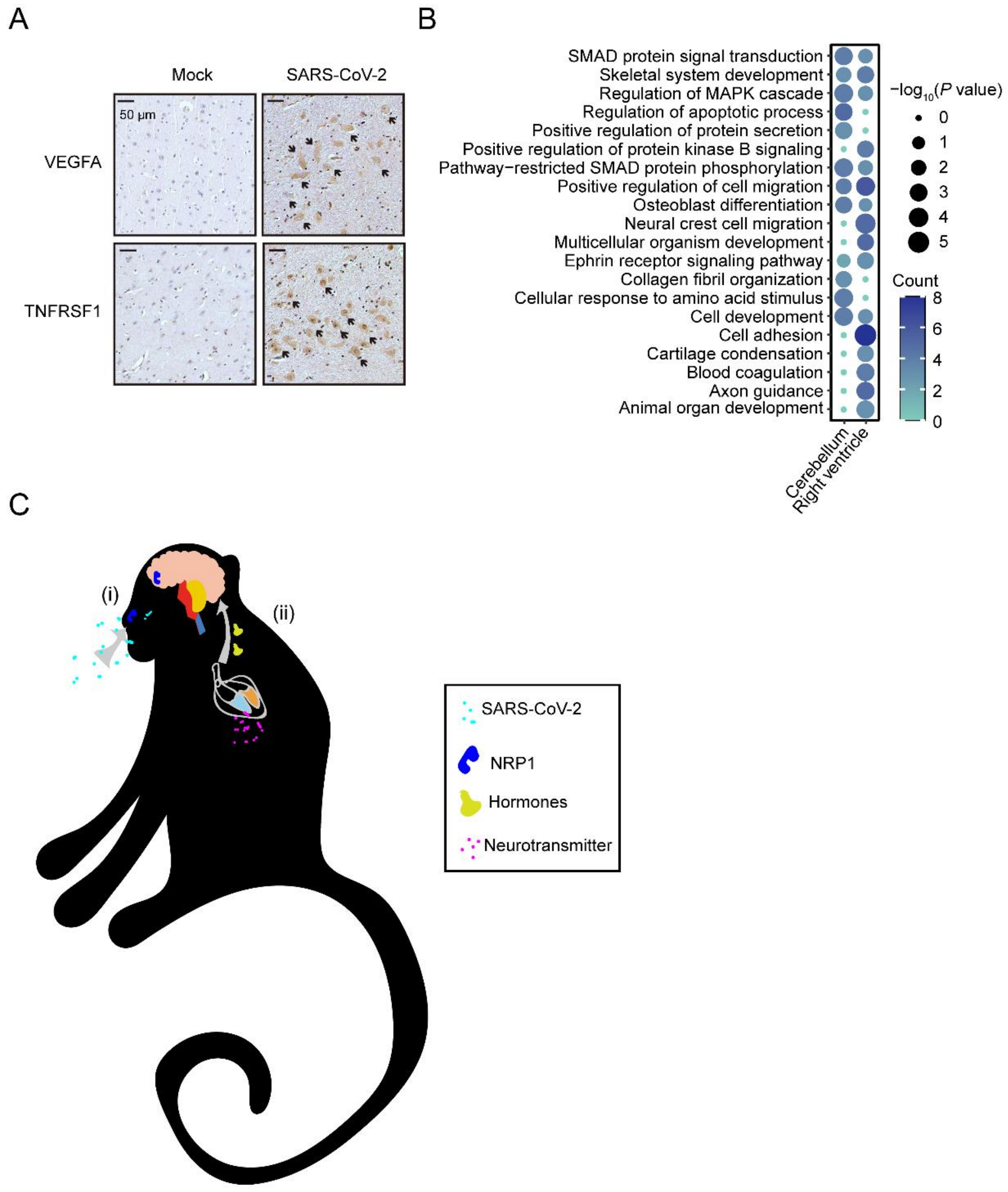
A
Mock
SARS-CoV-2
50 μm
VEGFA
TNFRSF1
B
SMAD protein signal transduction
Skeletal system development
Regulation of MAPK cascade
Regulation of apoptotic process
Positive regulation of protein secretion
Positive regulation of protein kinase B signaling
Pathway−restricted SMAD protein phosphorylation
Positive regulation of cell migration
Osteoblast differentiation
Neural crest cell migration
Multicellular organism development
Ephrin receptor signaling pathway
Collagen fibril organization
Cellular response to amino acid stimulus
Cell development
Cell adhesion
Cartilage condensation
Blood coagulation
Axon guidance
Animal organ development
Cerebellum
Right ventricle
−log10(P value)
0
1
2
3
4
5
Count
8
6
4
2
0
C
(i)
(ii)
SARS-CoV-2
NRP1
Hormones
Neurotransmitter
